# A Novel Long Non‐Coding RNA lnc030 Maintains Breast Cancer Stem Cell Stemness by Stabilizing SQLE mRNA and Increasing Cholesterol Synthesis

**DOI:** 10.1002/advs.202002232

**Published:** 2020-11-30

**Authors:** Yilu Qin, Yixuan Hou, Shuiqing Liu, Pengpeng Zhu, Xueying Wan, Maojia Zhao, Meixi Peng, Huan Zeng, Qiao Li, Ting Jin, Xiaojiang Cui, Manran Liu

**Affiliations:** ^1^ Key Laboratory of Laboratory Medical Diagnostics Chinese Ministry of Education Chongqing Medical University Chongqing 400016 China; ^2^ Experimental Teaching Center of Basic Medicine Science Chongqing Medical University Chongqing 400016 China; ^3^ Department of Surgery Samuel Oschin Comprehensive Cancer Institute Cedars‐Sinai Medical Center Los Angeles CA 90048 USA

**Keywords:** cancer stem cells, cholesterol synthesis, lnc030, PI3K/Akt signaling, SQLE

## Abstract

Cancer stem cells (CSCs) are considered the roots of cancer metastasis and recurrence (CSCs), due in part to their self‐renewal and therapy resistance properties. However, the underlying mechanisms for the regulation of CSC stemness are poorly understood. Recently, increasing evidence shows that long non‐coding RNAs (lncRNAs) are critical regulators for cancer cell function in various malignancies including breast cancer, but how lncRNAs regulate the function of breast cancer stem cells (BCSCs) remains to be determined. Herein, using lncRNA/mRNA microarray assays, a novel lncRNA (named lnc030) is identified, which is highly expressed in BCSCs in vitro and in vivo, as a pivotal regulator in maintaining BCSC stemness and promoting tumorigenesis. Mechanistically, lnc030 cooperates with poly(rC) binding protein 2(PCBP2) to stabilize squalene epoxidase (SQLE) mRNA, resulting in an increase of cholesterol synthesis. The increased cholesterol in turn actives PI3K/Akt signaling, which governs BCSC stemness. In summary, these findings demonstrate that a new, lnc030‐based mechanism for regulating cholesterol synthesis and stemness properties of BCSCs. The lnc030‐SQLE‐cholesterol synthesis pathway may serve as an effective therapeutic target for BCSC elimination and breast cancer treatment.

## Graphical abstract

Lnc030, a novel lncRNA, is highly expressed in BCSCs and acts as a pivotal regulator in maintaining BCSC stemness and tumorigenesis. Lnc030 cooperates with PCBP2 to stabilize SQLE mRNA, resulting in an increased cholesterol which actives PI3K/Akt signaling in governing BCSC stemness. The lnc030‐SQLE‐cholesterol synthesis pathway may serve as an effective therapeutic target for BCSC elimination and breast cancer treatment.

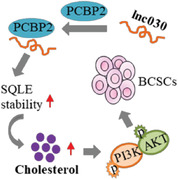

## 1 Introduction

Breast cancer (BC) is the most frequent malignancy in women worldwide.^[^ ^1^ ^]^ With the rapid development of treatment modalities, such as surgical resection, endocrine therapy, chemotherapy, and immunotherapy, the clinical outcomes of breast cancer have been improved.^[^ ^2^ ^]^ However, the majority of deaths in advanced breast cancers are due to distant metastasis or relapse.^[^ ^3^ ^]^ Therefore, effective measures are urgently needed to prevent and treat breast cancer metastases and relapses.

Previous studies have shown that there are many abnormally expressed protein‐encoding genes associated with breast cancers. However, protein‐coding genes account for only 2% of the human genome, whereas more than 90% of the human genome is transcribed into non‐coding RNA.^[^ ^4^ ^]^ Long non‐coding RNAs (lncRNAs), a subgroup of non‐coding RNAs, have more than 200 nucleotides and do not encode any protein.^[^ ^5^ ^]^ Increasing evidence has shown that lncRNAs play pivotal roles in various biological processes and disease progression, including metabolic remodeling, differentiation of embryonic stem cells and tumor initiation, growth, and metastasis.^[^ ^6^, ^7^, ^8^ ^]^ To date, little is known about the underlying mechanisms of lncRNAs regulating cancer stem cells (CSCs), including breast cancer stem cells (BCSCs).

CSCs possess unique abilities, including self‐renewal, differentiation, and drug‐resistance, all of which may contribute to distant metastasis or relapse of tumors.^[^ ^9^ ^]^ CSCs have been identified in many tumors including breast cancer. CD44+CD24‐ and ALDH+ are the most common markers of BCSCs.^[^ ^10^, ^11^ ^]^ Increased levels or activities of the ALDH enzyme indicate a high tumorigenic potential of cancer cells and the ability of CSC self‐renewal.^[^ ^12^ ^]^ Emerging evidence suggests targeting CSCs may be effective approach for anti‐cancer treatment.^[^ ^13^ ^]^ An in‐depth understanding of the mechanisms underlying the CSC stemness property is critical for eradicating CSC. However, it is unclear how BCSCs maintain their stemness. Recently, cancer metabolism has emerged as a potential diagnostic and therapeutic target. The metabolic properties of BCSCs and their link to CSC stemness are poorly understood.

Cholesterol is an essential component in various cells and has a key role in cell signaling. It is precursor for steroid hormones, bile acid, and other biological molecules. Abnormal changes of lipids have been detected in tumors, which provide cancer cells with building blocks, metabolic intermediates, and ATP for rapid cell proliferation and energy consumption. To date, cholesterol metabolism, as an important branch of lipid metabolism, has received much attention in cancer research. Recent reports show a pivotal link between cancer progression and cholesterol metabolism.^[^ ^14^ ^]^ There is an epidemiological correlation between blood cholesterol levels and tumor incidence or mortality.^[^ ^15^ ^]^ Intracellular cholesterol levels are elevated in tumor tissues and are crucial for cancer cell growth, migration, and invasion.^[^ ^16^ ^]^ CSCs also utilize cholesterol to maintain self‐renewal. For example, cholesterol enhances the stemness of colorectal CSCs through the MAPK signaling pathway.^[^ ^17^ ^]^ Another study indicated that cholesterol biosynthesis‐associated proteins are elevated in mammospheres compared with PDX tumors.^[^ ^18^ ^]^ Our unpublished proteomics and mRNA profiling also indicated that cholesterol synthesis was abnormal in breast cancer and BCSCs. All this evidence raises an interesting scientific question: abnormal cholesterol synthesis might play a role in maintaining the properties of CSCs. To date, how cholesterol is increased in CSCs and whether cholesterol plays an essential role in CSCs are still poorly understood.

In this study, we show that a new lncRNA, lnc030, is highly expressed in BCSCs and is required for maintaining BCSC stemness. Lnc030 interacts with poly(rC) binding protein 2 (PCBP2) to regulate SQLE mRNA stability, which promotes cellular cholesterol synthesis, leading to activation of the PI3K/AKT pathway for governing the stemness of BCSCs.

## 2 Results

### 2.1 Lnc030 Is Highly Expressed in BCSCs

**Figure 1:**
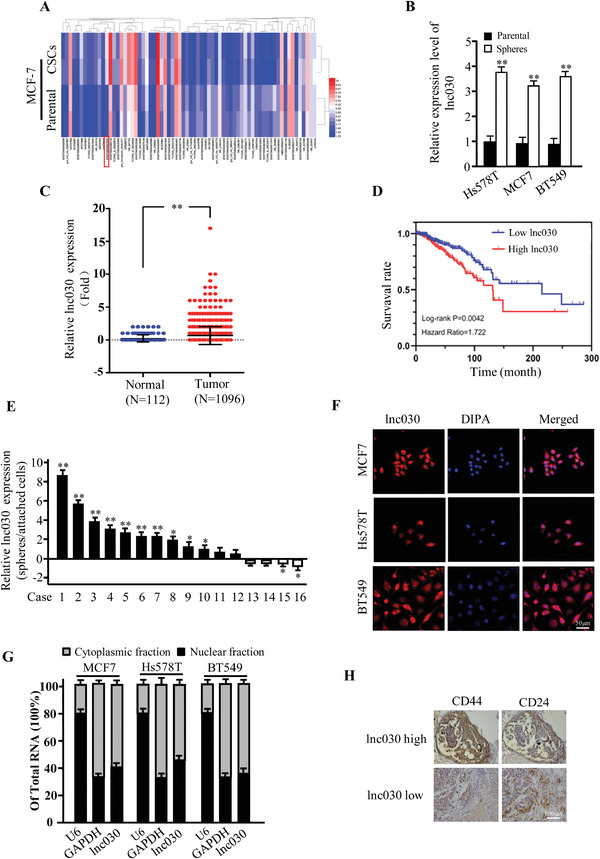
Lnc030 is highly expressed in BCSCs. A) Heatmap of lncRNA expression profiles between MCF‐7 and BCSC derived from epithelial‐mesenchymal transited MCF‐7 (MCF‐7/BCSC) using microarray assay. B) Lnc030 levels were evaluated in spheres and their parental BC cells by real‐time PCR (**p < 0.01). C) High level of lnc030 in breast tumor tissues (*n* = 1096) compared in adjacent normal breast tissues (*n* = 112) was identified using TCGA database (**p < 0.01). D) High level of lnc030 was correlated with a poor survival rate in TCGA database. E) The relative fold of lnc030 were evaluated in spheres and attached cells derived from randomly selected breast tumors by real‐time PCR (*n* = 16) (*p < 0.05, **p < 0.01). F) Lnc030 intracellular localization was visualized in breast cancer cells by RNA‐FISH assays. Representative images of lnc030 in Hs578T, MCF‐7, and BT549 cells are shown. DAPI: 4’,6‐diamidino‐2‐phenylindole; the used probe: lnc030 (Scale bar, 50 µm). G) Lnc030 fractionation in different breast cancer cells was followed by qRT‐PCR. U6 RNA serves as a positive control for nuclear gene expression. GAPDH serves as a positive control for cytoplasmic gene expression. Data are shown as means ± SD (*n* = 3). H) Representative images of IHC staining for CD44 and CD24 in breast tumor tissues with high or low lnc030 expression (Scale bar, 100 µm).

Previous studies indicated that epithelial‐mesenchymal transition (EMT) can endow cancer cells with stem cell‐like phenotypes.^[^ ^19^ ^]^ To investigate whether dysregulated lncRNAs are involved in BCSC formation, we performed lncRNA/mRNA microarray analysis to scan these dysregulated lncRNAs between MCF‐7 and epithelial‐mesenchymal transited MCF‐7 (named MCF‐7/BCSC). As shown in **Figure** **1**A, with a cut‐off fold change >2.0 and *p* < 0.05, a set of dysregulated lncRNAs between MCF‐7 and MCF‐7/BCSC cells were identified. It was found that 29 lncRNAs were up‐regulated, while 9 lncRNAs were down‐regulated, in MCF‐7/BCSCs compared to MCF‐7 (Figure 1A). Ten randomly chosen lncRNAs were validated in MCF‐7 and MCF‐7/CSCs (Figure S1A, Supporting Information). After being analyzed by bio‐informatics, the up‐regulated novel lncRNA ENST00000523030.1 (named lnc030, gene_id AC009908.1/LOC105375744) was chosen for further investigation due to its functional association with cholesterol metabolism. Lnc030 is located in human chromosome 8q24.13 and is a 412nt transcript with 2 exons (Figure S1B, Supporting Information), which has no coding potential evaluated by online bioinformatics analysis (http://cpc.cbi.pku.edu.cn) (Figure S1C, Supporting Information). Lnc030 was highly expressed in CSC mammospheres derived from Hs578T and BT549 cells and moderately expressed in CSC mammospheres derived from MCF‐7 as compared to their parental cells (Figure 1B). Indeed, the increased lnc030 expression was detected in the majority of breast tumor tissues (*N* = 1096) in comparison with normal tissues (*N* = 112) using the TCGA database (Figure 1C). High expression of lnc030 indicated a poor prognosis in breast cancer patients from TCGA database (Figure 1D). In addition, we also found that lnc030 expression was significantly increased in breast cancer spheroids than that in attached primary cells isolated from our cohort of patients with breast cancer (Figure 1E). Using RNA fluorescence in situ hybridization (RNA‐FISH) (Figure 1F) and cellular fractionation assays (Figure 1G), we found that lnc030 was mainly localized in the cytoplasm of Hs578T, BT549, and MCF‐7 breast cancer cells. Moreover, we found that the breast cancer tissues with high levels of lnc030 had higher expression of CD44 and lower expression of CD24 compared with these with low levels of lnc030 (Figure 1H), and high levels of lnc030 were also closely associated with pathological characteristics of tumors, such as lymph node metastasis and pathological grade (Table S1, Supporting Information). Taken together, these data demonstrate that lnc030 is highly expressed in BCSCs and closely is associated with breast cancer malignancy.

### 2.2 Lnc030 Is Required for Maintaining BCSC Stemness

**Figure 2:**
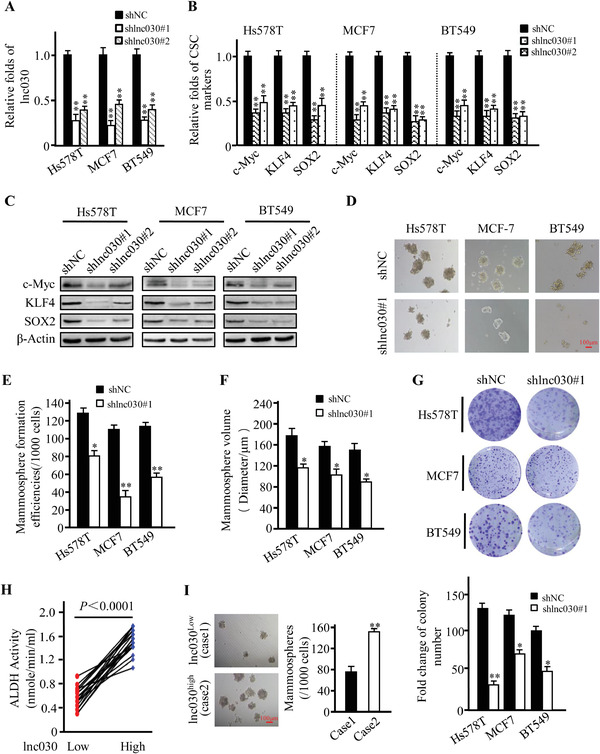
Lnc030 is required for maintaining BCSCs' stemness. A) Lnc030 silenced efficiencies by two independent shRNAs were evaluated in BCSCs. Data are shown as means ± SD (**p < 0.01). B,C) The expression of pluripotent transcription factors c‐Myc, KLF4, and SOX2 were analyzed in BCSC with shlnc030 or shNC by qRT‐PCR (B) and Western blotting (C). Data are shown as means ± SD (**p < 0.01). D) Spheres‐formation abilities were tested in lnc030‐knocked down BC cells and their control cells (Scale bar, 100 µm). E,F) The mammosphere‐formation efficiencies (E) and spheres sizes (F) were quantified from (D). The data represent means ± SD (*n* = 3, *p < 0.05, **p < 0.01). G) Representative images of colonies (upper panel) derived from the lnc030‐knocked down and control cells are shown. The numbers of colonies (down panel) were counted. The data represent means ± SD (*p < 0.05, **p < 0.01). H) ALDH activity in breast tumor tissues with low or high lnc030 was measured (*n* = 40, *p* < 0.0001). I) mammosphere‐formation abilities were measured using the tumor cells isolated from lnc030 low expression cases (#1) and lnc030 high expression cases (#2) (**p < 0.01, Scale bar, 100 µm).

To examine the role of lnc030 in retaining BCSC stemness, we knocked down lnc030 in Hs578T, BT549, and MCF7 using two independent lentivirus‐mediated short hairpins RNAs (shRNAs) (**Figure** **2**A). We also overexpressed ectopic lnc030 in Hs578T, BT549, and MCF7 cells (Figure S1D, Supporting Information). As expected, lnc030 knockdown with two independent shRNAs significantly decreased the expression of pluripotent transcription factors such as c‐Myc, KLF4, and SOX2 compared with their control cells (shNC) (Figure 2B,C), whereas ectopic lnc030 overexpression significantly elevated the expression of these factors (Figure S1E,S1F, Supporting Information). We then chose shlnc030#1 for the following experiment. Importantly, mammosphere formation efficiency was dramatically reduced in lnc030‐knockdown BC cells (Figure 2D–F) and enhanced in lnc030‐overexpressing BC cells (Figure S2A–S2C, Supporting Information). Additionally, knockdown of lnc030 markedly impaired colony formation, while ectopic lnc030 increased colony formation of BC cells (Figure 2G and Figure S2D, Supporting Information). Moreover, immunofluorescence staining also confirmed that the stemness markers such as c‐Myc and CD44 were significantly reduced in lnc030‐silenced spheres (Figure S2E, Supporting Information). Flow cytometry analysis showed that CD44^+^/CD24^−/low^ cell populations were decreased in lnc030‐knockdown Hs578T, MCF7, and BT549 derived spheres compared with control spheres (Figure S2F, Supporting Information). We also detected activity of acetaldehyde dehydrogenase (ALDH) in sets of breast tumor tissues. Indeed, increased ALDH activity was detected in lnc030‐high breast cancer tissues compared with that in lnc030‐low tumor tissues (Figure 2H), and the breast cancer cells isolated from breast tumor with high lnc030 expression showed higher mammosphere‐formation abilities in comparison to those with low lnc030 expression (Figure 2I). These results suggest that lnc030 plays a crucial role in maintaining BCSCs' stemness.

### 2.3 Lnc030 Promotes SQLE Expression to Maintain BCSCs Stemness

**Figure 3:**
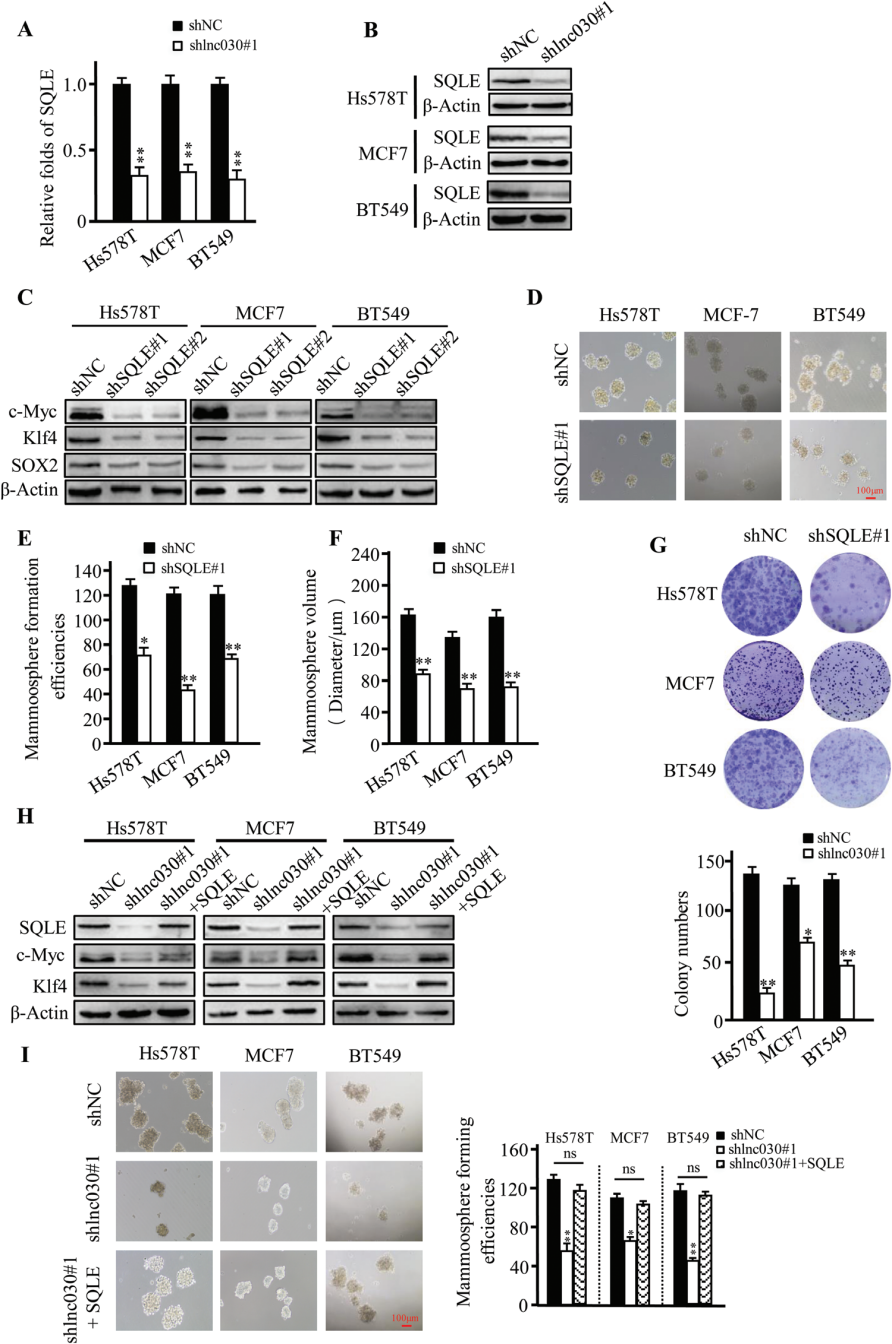
Lnc030 promotes SQLE expression to maintain BCSCs' stemness. A, B) mRNA (A) and protein levels (B) of SQLE in lnc030‐knockdown BCSCs and control BCSCs were determined by qRT‐PCR and Western blotting. The data represent means ± SD (**p < 0.01). C) The CSC markers, c‐Myc, KLF4, SOX2 protein levels were detected in SQLE‐knockdown BCSCs and control BCSCs. D–F). Sphere‐formation abilities were tested in SQLE‐knockdown and control BCSCs (Scale bar, 100 µm). The sphere‐formation efficiency (E) and spheres sizes (F) were quantified from (D). The data represent means ± SD (*n* = 3, *p < 0.05, **p < 0.01). G) Representative images of colonies (upper panel) derived from the lnc030‐knockdown and control cells are shown. The numbers of colonies (down panel) were counted. The data represent means ± SD (*p < 0.05, **p < 0.01). H) SQLE, c‐Myc, and KLF4 proteins were determined by Western blotting in lnc030‐knockdown BCSCs, BCSCs with lnc030‐knockdown and ectopic SQLE, and control BCSCs. I) Sphere‐formation efficiency was tested in lnc030‐knockdown, lnc030 knockdown combined with ectopic SQLE, and their control of BC cells. The data represent means ± SD (*n* = 3, *p < 0.05, **p < 0.01, Scale bar, 100 µm).

LncRNA can participate in tumor development by regulating its neighboring genes.^[^ ^20^ ^]^ To analyze whether lnc030 regulates its neighboring gene, squalene epoxidase (SQLE) (Figure S3A, Supporting Information), we first evaluated whether knockdown or overexpression of lnc030 impacts SQLE expression. Indeed, lnc030 knockdown significantly decreased SQLE expression (**Figure** **3**A,B), while ectopic lnc030 expression increased SQLE expression (Figure S3B,S3C, Supporting Information). Next, we asked whether SQLE could maintain the stemness of BCSCs. Silence of SQLE using two independent lentivirus‐mediated shRNAs (Figure S3D,S3E, Supporting Information) led to notable decrease of the pluripotent transcription factors c‐Myc, KLF4, and SOX2 (Figure 3C and Figure S3F, Supporting Information). We then chose shSQLE#1 for the following experiment. Correspondingly, knockdown of SQLE dramatically reduced sphere‐formation efficiency and sphere sizes (Figure 3D–F). In addition, as indicated in Figure 3G, colony formation was significantly reduced by silencing SQLE expression in BC cells. To further confirm whether lnc030 maintains BCSCs stemness by regulating SQLE, we restored SQLE expression in the lnc030‐knockdown cells. Interestingly, SQLE restoration could rescue the expressions of pluripotent transcription factors c‐Myc, KLF4, and SOX2 (Figure 3H). The reduced potentials of mammosphere formation in lnc030‐silenced BC cells were partially rescued by ectopic SQLE expression in these BC cells (Figure 3I).

Some reports indicated that SQLE is highly expressed in breast cancer tissues than adjacent normal tissues, and high levels of SQLE are associated with distant metastasis‐free survival.^[^ ^21^, ^22^ ^]^ To investigate the clinical significance of SQLE, we examined expression of lnc030, SQLE, and c‐Myc in breast tumor tissues and the potential correlation among lnc030, SQLE, and c‐Myc in clinic patients with breast cancer. Obviously, high levels of SQLE were closely related with lnc030 high expression (Figure S3G, Supporting Information), and a positive correlation was also unraveled between SQLE and c‐Myc expression in breast tumors (Figure S3H, Supporting Information). These data suggest that lnc030 may be involved in BCSC stemness maintenance via regulating SQLE.

### 2.4 Lnc030 Cooperates with PCBP2 to Increase SQLE mRNA Stability

**Figure 4:**
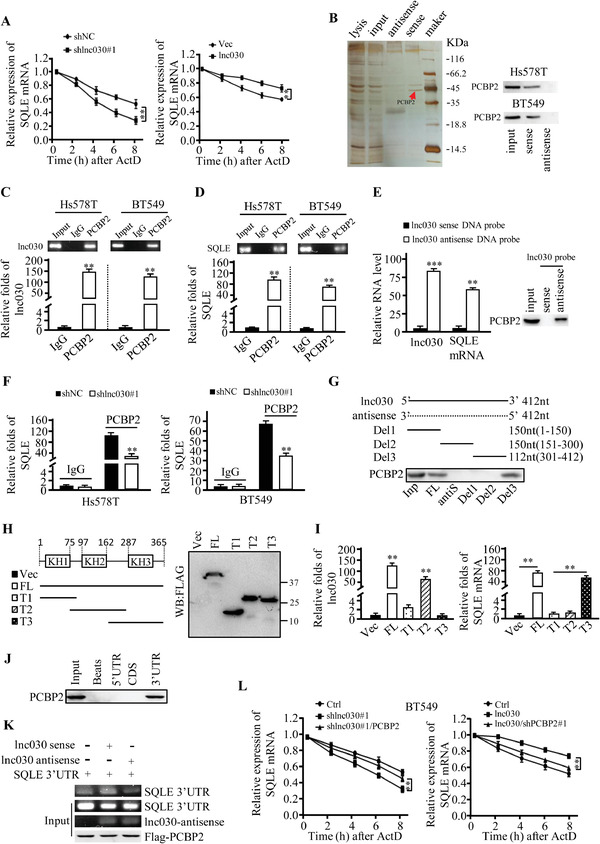
Lnc030 cooperates with PCBP2 to increase SQLE mRNA stability. A) Lnc030‐knockdown, Lnc030‐overexpressing breast cancer cells, and their control cells were incubated with actinomycin D (2.5 µg mL^−1^) for the indicated periods of time. SQLE mRNA stability was determined by qRT‐PCR. Data are shown as means ± SD (*n* = 3, **p < 0.01, shlnc030 versus shNC, or lnc030 versus vector). B) RNA pull‐down was performed using sense and antisense lnc030, a significantly changed band of PCBP2 is shown. C, D) RIP assays were performed with spheres from Hs578T and BT549 cells using anti‐PCBP2. IgG served as the negative control. The indicated RNA enriched in the RIP precipitates using antibody anti‐PCBP2 or IgG was analyzed by qRT‐PCR. The data represent means ± SD (**p < 0.01). E) BT549 sphere lysates were incubated with either sense or antisense biotin‐labeled probe against lnc030 for the pull‐down assay. qRT‐PCR or Western blotting were used to detect the SQLE mRNA and PCBP2 protein in the pull‐down precipitates. Data shown are the mean ± SD (*n* = 3, **p < 0.01, ***p < 0.001). F) The enrichment of SQLE mRNA was assessed by RIP‐qPCR in spheres derived from lnc030‐silenced Hs578T and BT549. IgG served as the negative control. G) Western blot of PCBP2 in the precipitates using biotinylated full‐length, antisense, or truncated lnc030 as probe in pull‐down assay in BT549 (Inp: input; FL: full length of lnc030; antiS: antisense; Del: lnc030 deletion mutant.). H) Schematic structures of PCBP2 and deletion mappings of PCBP2 (Vec: vector; FL: full length of PCBP2; T: truncated fragment of PCBP2, and T1:1‐96; T2:76‐286; T3:163‐365). I) RIP‐qPCR to identify the lnc030 and SQLE binding domains in PCBP2 using full length or truncated PCBP2 protein (**p < 0.01). J) RNA pull‐down assay was performed using SQLE 5’‐UTR probe, CDS probe, or 3’‐UTR probe in BT549. PCBP2 protein was viewed by Western blot in the indicated precipitates of pull‐down assay. K) Purified recombinant Flag‐PCBP2 by M2 beads was incubated with in vitro‐synthesized lnc030 sense or antisense probe, and SQLE mRNA 3’UTR in the indicated combination. The bead‐bound RNAs were then eluted for RT‐PCR assay. L) Degradation rates of SQLE mRNA in BT549 transfected with sh lnc030#1, sh lnc030#1 combined with ectopic PCBP2, ectopic lnc030, ectopic lnc030 combined with shPCBP2#1, or control vector are evaluated by qRT‐PCR. The data represent means ± SD (**p < 0.01, sh lnc030#1 versus sh lnc030#1/PCBP2 or control, oe lnc030 versus lnc030/shPCBP2#1 or control).

We next asked how lnc030 increases SQLE expression. Because both of SQLE (Figure S3I, Supporting Information) and lnc030 (Figure 1F) located in the cytoplasm, we wondered whether lnc030 could regulate SQLE mRNA stability as most cytoplasm lncRNAs has done. Hs578T cells with lnc030 knockdown or overexpression, which were treated with actinomycin D for different periods of time, were used to assess the decline of SQLE mRNA levels. Knockdown of lnc030 caused a marked drop in the half‐life of SQLE mRNA, whereas overexpression of lnc030 substantially increased its half‐life (**Figure** **4**A). Considering that there is no complementary sequence between lnc030 and SQLE mRNA, we guessed lnc030 might regulate SQLE mRNA stability by interaction with a certain RNA binding protein (RBP), as other lncRNAs performed.^[^ ^23^ ^]^ Using RNA pull‐down, SDS‐PAGE, and mass spectrometry analysis, we identified the potential RBP, poly(rC) binding protein 2 (PCBP2), which was co‐precipitated with in vitro synthesized lnc030 but not with antisense lnc030 (Figure 4B). Furthermore, the RIP assays using an antibody against PCBP2 confirmed that PCBP2 was indeed enriched with lnc030 in Hs578T or BT549 spheres (Figure 4C,D). To further confirm that PCBP2 could bridge lnc030 and SQLE mRNA, we first performed pull‐down assay using a biotin‐labeled antisense DNA probe specifically against lnc030. Our data showed that SQLE mRNA and PCBP2 could be detected in the pull‐down precipitate (Figure 4E). Then, we tried to pull down SQLE mRNA using a biotinylated antisense DNA probe against SQLE mRNA, lnc030, and PCBP2 were co‐precipitated (Figure S3J, Supporting Information). These data suggested that PCBP2 acts as a mediator for lnc030 and SQLE mRNA interaction. In addition, knockdown of lnc030 greatly reduced the binding between PCBP2 and SQLE mRNA in the RIP complex (Figure 4F). Next, to locate the specific fragment of lnc030 binding with PCBP2, we performed deletion‐mapping analysis and RNA pull‐down. The results showed that lnc030 fragment 301–412nt (Del3) could bind with PCBP2 (Figure 4G). PCBP2 consists of 3K homology (KH) domains. Flag‐tagged full‐length and three truncated fragments of PCBP2 overlapping with each other on the inactive regions were established (Figure 4H). RIP assays revealed that lnc030 is responsibly bound to the KH2 (T2) of PCBP2 (Figure 4I, left panel), and SQLE is mainly bound to the KH3 domain (T3) (Figure 4I, right panel). It has been recently shown that PCBP2 could stabilize STAT1 mRNA through binding to its 3’UTR.^[^ ^24^ ^]^ Therefore, we wondered whether PCBP2 could regulate mRNA stability of SQLE by binding to 3’‐UTR of SQLE. Performed RNA pull‐down assay with in vitro transcript probes of SQLE mRNA, we found that PCBP2 was specifically pulled down by SQLE mRNA probe containing 3’‐UTR, but not 5’‐UTR or CDS regions (Figure 4J). In addition, an in vitro binding assay demonstrated that lnc030, rather than its antisense RNA, could increase the binding between PCBP2 and SQLE 3’‐UTR (Figure 4K). These data demonstrated that lnc030, PCBP2, and SQLE 3’‐UTR can form a ternary complex, and lnc030 promotes the interaction between PCBP2 and SQLE. Knockdown of lnc030 had no significant impact on PCBP2 expression at either RNA or protein level (Figure S3K, Supporting Information), suggesting PCBP2 merely serves as a mediator for the binding between lnc030 and SQLE. To further confirm that lnc030 could promote SQLE mRNA stability via PCBP2, we silenced PCBP2 (Figure S4A,S4B, Supporting Information), and SQLE mRNA half‐life was evaluated under treatment with actinomycin D. As shown in Figure 4L and Figure S4C, Supporting Information, knockdown of lnc030 caused a significant decrease in SQLE mRNA half‐life, which could be blunted by ectopic expression of PCBP2; however, lnc030 overexpression failed to increase SQLE mRNA half‐life in PCBP2‐knockdown cells. In accordance, ectopic lnc030 led to increased SQLE in both mRNA and protein level, but not in PCBP2‐knockdown spheres (Figure S4D,S4E, Supporting Information). Additionally, knockdown of lnc030 in spheres reduced mRNA and protein expressions of SQLE, which could be rescued by ectopic PCBP2 (Figure S4F, Supporting Information). Collectively, these results demonstrate that stabilizing effect of lnc030 on SQLE mRNA is dependent on PCBP2. Next, we determined whether lnc030 could maintain BCSCs stemness via PCBP2. Indeed, silence of PCBP2 in BC cells mitigated ectopic lnc030‐caused increases of c‐Myc and KLF4, and mammosphere formation efficiency (Figure S4G,S4H, Supporting Information). Taken together, these data demonstrate that lnc030 regulates SOLE mRNA stability through interaction with PCBP2.

### 2.5 Intracellular Cholesterol Promotes Maintenance of BCSC Stemness

**Figure 5:**
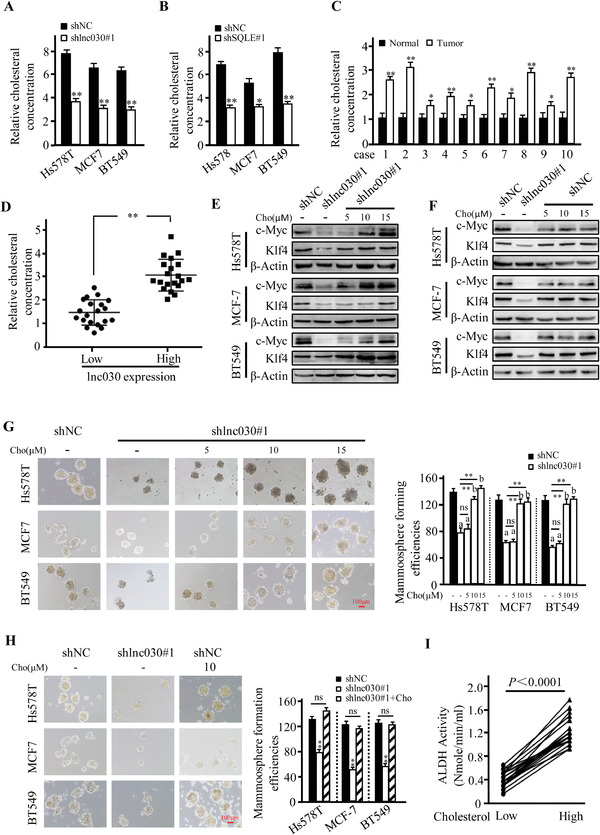
Intracellular cholesterol involves in maintenance of BCSC stemness. A,B) Intracellular cholesterol was measured in lnc030‐knockdown (A) or SQLE‐knockdown (B) Hs578T, BT549, MCF‐7 derived spheres and their control spheres. Data are shown as means ± SD (*p < 0.05, **p < 0.01). C) Cholesterol amount in the randomly selected breast tumor tissue and their matched non‐tumor tissues were measured. Data are shown as means ± SD (*n* = 10, * *p* < 0.05, ** *p* < 0.01). D) The cholesterol concentration in lnc030 high or low expression BC tissues were measured. Data are shown as means ± SD (*n* = 40, ** *p* <0.01). E, F) c‐Myc and KLF4 proteins were detected by Western blotting in lnc030‐knockdown (E) and control BCSCs (F) under treatment with or without cholesterol (labeled Cho) at 5, 10, or 15 µM for 48 h. G,H) Sphere‐formation potentials were detected for the Lnc030‐knockdown and control breast cancer cells. Lnc030‐silenced cells were treated with cholesterol at 5, 10, or 15 µM for every 3 days in the suspend culture G) (a, *p* < 0.01, versus shNC + vehicle; b, *p* > 0.05, versus shNC + vehicle; ns, *p* > 0.05, versus sh lnc030#1 + vehicle; ** *p* < 0.01, versus sh lnc030#1 + vehicle; Scale bar, 100 µm); the control cells were treated with the optimum concentration (10 µM) of cholesterol(H) (**p < 0.01, sh lnc030 versus shNC; Scale bar, 100 µm). I) ALDH activity in breast cancer tissues with low or high levels of cholesterol was detected (*n* = 40, *p* < 0.0001).

SQLE is a key rate‐limiting enzyme for cholesterol synthesis, and cholesterol biosynthesis can activate Notch signaling to promote hematopoietic stem and progenitor cell (HSPC) emergence.^[^ ^25^ ^]^ We wondered whether lnc030 could regulate SQLE expression and cholesterol synthesis to maintain BCSCs' stemness. First, we measured intracellular cholesterol levels in lnc030‐ or SQLE‐knockdown spheres and their control spheres. Indeed, knockdown of lnc030 or SQLE significantly decreased the levels of intracellular cholesterol compared with the control spheres (**Figure** **5**A,B). Excitingly, a high concentration of cholesterol was detected in breast tumor tissues than the matched normal tissues (Figure 5C). More importantly, high concentrations of cholesterol were found in lnc030^high^ breast tumors in comparison with lnc030^low^ tumors (Figure 5D). To demonstrate the effects of cholesterol on BCSC stemness maintenance, we treated BCSCs with cholesterol at different concentrations, and expression of the pluripotent transcription factors c‐Myc and KLF4 was assessed. Cholesterol could restore the expression of pluripotent transcription factor c‐Myc and KLF4 in lnc030‐knockdown BCSCs in a dose‐dependent manner, 10 µM of cholesterol could exert its effect on pluripotent transcription factor expression in lnc030‐knockdown BCSCs (Figure 5E) rather than in control BCSCs (Figure 5F). Similarly, SQLE‐silence mediated inhibitory effects on the expression of c‐Myc and KLF4 in BCSC were abolished by treatment with exogenous cholesterol (Figure S5A, Supporting Information), but exogenous cholesterol had no much effects on the expression of c‐Myc and KLF4 for control BCSCs (Figure S5B, Supporting Information). Correspondingly, cholesterol could rescue the mammosphere‐formation efficiency of lnc030‐knockdown (Figure 5G) or SQLE‐silenced BCSCs (Figure S5C, Supporting Information) rather than control BCSCs (Figure 5H, and Figure S5D, Supporting Information). And moreover, the concentration of intracellular cholesterol was positively related with ALDH activity in clinic tumors (Figure 5I). These data demonstrate that lnc030‐mediated cholesterol increase in breast tumors promotes BCSC formation and stemness maintenance.

### 2.6 Lnc030 Maintains BCSCs Stemness through PI3K/Akt Signaling

**Figure 6:**
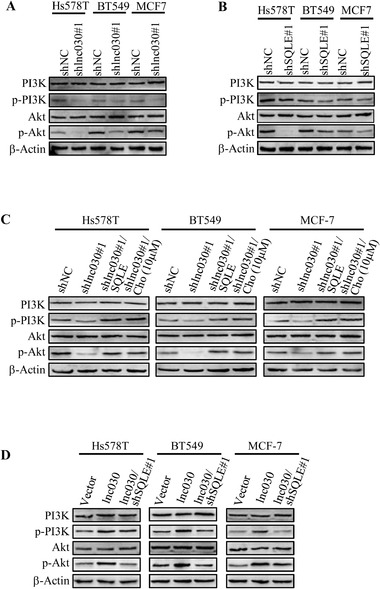
Lnc030 maintains BCSCs stemness through PI3K/Akt signaling. A, B) Western blotting was used to evaluate the activation of PI3K/Akt signaling in BCSC spheres derived from BC cells with sh lnc030#1 (A), shSOLE#1 (B), and shNC. (C). Lnc030‐silenced breast cancer cells were transfected with ectopic SOLE or treated with cholesterol (10 µM), activation of PI3K/Akt signaling was evaluated by Western blotting in these tested and control BCSC spheres. D) Hs578T, BT549, and MCF‐7 transfected with lnc030, or lnc030 combined with shSQLE#1, the activation of PI3K/Akt signaling was detected by Western blotting in BCSC spheres.

Cholesterol has been reported to regulate several oncogenic signals, such as PI3K/Akt and Wnt/*β*‐catenin.^[^ ^26^, ^27^, ^28^ ^]^ PI3K/Akt, Wnt/*β*‐catenin, and Notch/YAP are involved in self‐renewal of CSCs. To investigate the downstream molecular mechanisms underlying the oncogenic function of lnc030, we examined these CSCs‐related oncogenic signals in lnc030‐knockdown BCSCs and their control BCSCs. We found that Wnt/*β*‐catenin, Notch/YAP, and NF‐*κ*B signaling has no significant change in the lnc030‐knockdown BCSCs in comparison with their control BCSCs (Figure S5E, Supporting Information). Interestingly, silence of lnc030 markedly reduced the levels of phosphorylated PI3K (p‐PI3K) and phosphorylated Akt (p‐Akt) (**Figure** **6**A). In addition, phosphorylated PI3K (p‐PI3K) and Akt (p‐Akt) were also decreased in the SQLE‐knockdown BCSCs compared to their control BCSCs (Figure 6B), suggesting that PI3K/Akt signaling is downstream of lnc030‐SQLE signaling axis. To further confirm the effect of lnc030/SQLE signaling on the PI3K/Akt pathway, ectopic SQLE was transfected into lnc030‐knockdown BCSCs, or 10 µM of cholesterol was used to treat the lnc030‐knockdown BCSCs. Ectopic SQLE or cholesterol treatment could restore the expression of p‐PI3K and p‐Akt in lnc030‐knockdown BCSCs (Figure 6C). Although ectopic lnc030 in SQLE‐wild type BCSCs could enhance the expressions of p‐PI3K and p‐Akt, but failed to increase their levels in SQLE‐knockdown BCSCs (Figure 6D), indicating that SQLE is a mediator for lnc030 to activate PI3K/Akt signaling. These data demonstrate that the lnc030/SQLE signal axis maintains BCSCs stemness through the PI3K/Akt signaling pathway.

### 2.7 Lnc030‐SQLE‐Cholesterol Promotes Breast Cancer Initiation and Progression

**Figure 7:**
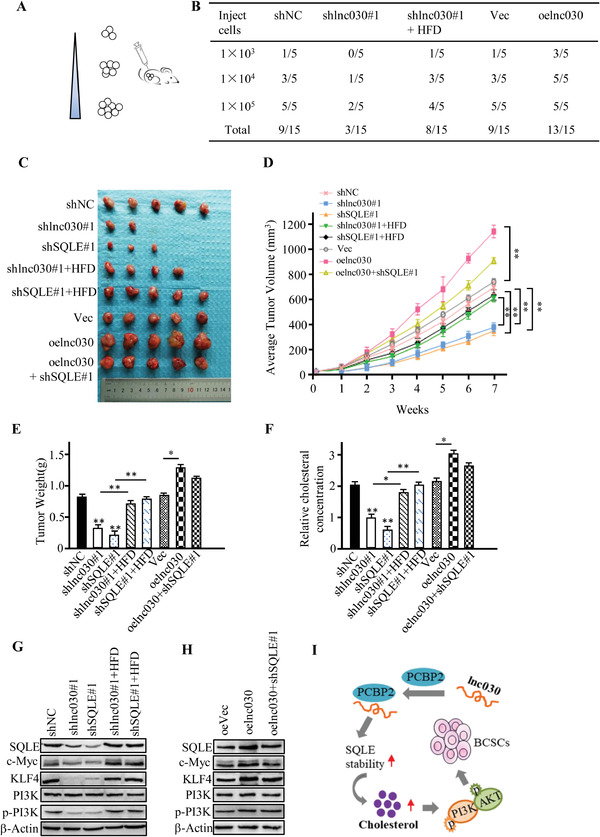
Lnc030 Promotes Breast Cancer Initiation and Progression. A) Schematic diagram to show in vivo xenograft experiments of BCSC limiting dilution assay. B) limiting dilution assay of indicated MCF7 BCSCs (1 × 10^3^, 1 × 10^4^, 1 × 10^5^/mouse) was used to investigate the impacts of lnc030 on tumor‐initiation (*n* = 5 for each mice group). C–E). The indicated engineered BT549 or control cells (2 × 10^6^/mouse) were injected into mammary fat pad, and representative pictures of tumors (C), tumor growth curve (D), and tumor weight (E) of each mouse group were measured. The data represent means ± SD (* *p* < 0.05; ** *p* < 0.01). F) Cholesterol concentrations in mice tumors of each experimental group were measured. The data represent means ± SD (* *p* < 0.05; ** *p* < 0.01). G,H) Protein levels of SQLE, PI3K, p‐PI3K, and CSC‐associated c‐Myc and KLF4 in tumors were evaluated by Western blotting. I) A schematic diagram to depict the role of lnc030 in maintenance of BCSC stemness through regulating SQLE and cholesterol accumulation to stimulate PI3K/Akt signaling.

Finally, we tested the pro‐tumorigenesis function of CSC‐related lnc030 in vivo. Thus, a limiting dilution assay was performed (**Figure** **7**A). As shown in Figure 7B, lnc030 knockdown significantly reduced tumor‐initiating capacity of spheroids in nude mice, and feeding mice with high fat/cholesterol diet (HFD) could rescue the tumor‐initiating capacity of lnc030‐knockdown BCSC; correspondingly, lnc030 overexpression in CSCs increased their tumor‐initiating capacity in mice. In addition, lnc030 or SQLE knockdown attenuated tumor growth, tumor size, and tumor weight of xenograft in vivo; cholesterol feeding fueled tumor growth in mice xenografts injected with lnc030‐knockdown or SQLE‐silenced breast cancer cells; and ectopic lnc030 in breast cancer cells notably facilitated tumor growth, and knockdown of SQLE in lnc030‐overexpressing tumor cells attenuated tumor growth (Figure 7C–E). Consistently with the concentration of cholesterol in tumor tissues of xenografts, the SQLE, phosphorylated PI3K, and CSC‐associated c‐Myc and KLF4 proteins displayed the corresponding changes in line with the tumor growth in each group of xenografts (Figure 7F–H). And IHC staining of SQLE, c‐Myc, KLF4 displayed similar changes in xenograft tumors (Figure S7A, Supporting Information). Taken together, our works validate that lnc03‐SQLE‐cholesterol signaling stimulates activation of PI3K/Akt, which is involved in BCSC stemness maintenance and fuels breast tumor initiation and tumor growth (Figure 7I). This work also suggests that targeting CSC‐specific lncRNA (e.g., Lnc030) and its downstream signaling may be an effective therapeutic option.

## 3 Discussion

CSCs harbor stem cell properties, such as self‐renewal, differentiation, and drug‐resistance. Metastasis and relapse in breast cancer patients can be attributed to breast CSCs (BCSCs). Therefore, interference of BCSCs may improve prognosis of patients with breast cancer. BCSCs own unique biomarkers, CD44^+^CD24^−/low^ and ALDH expression or activity have been known as common biomarkers of BCSCs.^[^ ^10^, ^11^ ^]^ However, the mechanism of BCSCs maintaining self‐renewal and stemness remains largely unclear. In the current study, we isolated the CD44^+^CD24^−^ subpopulation cells from breast cancer (BC) cells and BC tumors, and confirmed that lnc030, a novel lncRNA, plays a pivotal role in the self‐renewal and maintenance of BCSCs. It is known that some key pathways are involved in regulating CSC characteristics, which include PI3K/Akt, *β*‐catenin/Wnt, Notch, NF‐*κ*B.^[^ ^29^, ^30^ ^]^ Herein, we demonstrate lnc030 regulates self‐renewal and stemness of BCSCs through PI3K/Akt signal cascade.

LncRNAs participate in gene regulation and other cellular processes through various mechanisms, including recruiting chromatin complex, paring with other RNAs, and interacting with proteins.^[^ ^31^, ^32^, ^33^ ^]^ Studies have reported the regulatory role of lncRNAs in various tumors including breast cancer.^[^ ^34^, ^35^, ^36^ ^]^ It is noted that lncRNAs could regulate self‐renewal of embryonic stem cells.^[^ ^37^ ^]^ Recently, there are a few reports indicating that lncRNAs are involved in maintaining CSC stemness. For example, lncTCF7 was reported to regulate TCF7 expression by recruiting SWI/SNF complex and to activate Wnt signaling pathway in maintaining self‐renewal of liver cancer stem cells.^[^ ^38^ ^]^ In female esophageal carcinoma, exosomal FMRA‐AS1 facilitates maintenance of cancer stem‐like cells dynamic equilibrium via TLR7/NF‐*κ*B/c‐Myc signaling.^[^ ^39^ ^]^ LncRNA‐Hh boosts BCSC generation through the hedgehog signal pathway.^[^ ^40^ ^]^ However, the effects of lncRNAs on CSCs, especially BCSCs remain largely unknown. In this study, using lncRNA/mRNA microarray analysis, we identified a novel lnc030, which is highly expressed in BCSCs. More importantly, we found that lnc030 plays its functional role in stemness maintenance through stabilizing SQLE mRNA and increasing cholesterol synthesis in breast cancer. Additionally, high levels of lnc030 indicate a poor prognosis in breast cancer patients.

The functions of lncRNAs mainly depend on their subcellular distribution. Nuclear lncRNAs are combined with chromatin‐remodeling complexes to regulate its target gene expression at the transcriptional level;^[^ ^38^ ^]^ however, cytoplasmic lncRNAs, which act as ceRNAs or associate with protein to regulate the stability of mRNA or prevent protein degradation.^[^ ^32^, ^41^, ^42^ ^]^ In this study, we confirmed that lnc030 largely localizes in cytoplasma and contributes to SQLE mRNA stability. Although there is no direct binding site between lnc030 and SQLE, an RNA binding protein, PCBP2 was identified to play a mediator for lnc030 function. Lnc030, PCBP2, and SQLE were found to form a ternary complex to regulate SQLE stability, It was reported that lncRNA *lnc13* sponges with PCBP2 increasing *STAT1* mRNA expression to modulate pancreatic *β* cell inflammation.^[^ ^41^ ^]^ Here, we found that interaction between lnc030 and PCBP2 possesses a new function in self‐renewal of BCSCs.

SQLE is called squalene epoxidase, which acts as an oncogenic role in tumors, including breast cancer.^[^ ^43^, ^44^ ^]^ Ectopic expression of SQLE drives NAFLD‐induced hepatocellular carcinoma and becomes a potential pharmacological target.^[^ ^45^ ^]^ In breast cancer, CASIMO1 has been reported to interact with SQLE impacting the cell's metabolic homeostasis.^[^ ^46^ ^]^ In small cell lung cancer, SQLE binds to microRNAs and affects downstream target gene expression.^[^ ^47^ ^]^ However, the function of SQLE on CSCs has not been elucidated. In this study, we showed that SQLE can promote the maintenance of BCSCs' stemness. Interestingly, SQLE is a key rate‐limiting enzyme for cellular cholesterol synthesis. The synthesized cholesterol can be used for many building blocks, such as steroid hormones, and the metabolic intermediates may also be utilized for other biological processes. Importantly, we reveal that lnc030‐mediated stability of SQLE mRNA regulates SQLE protein expression and cholesterol synthesis, thus contributing to maintenance of BCSCs stemness.

Metabolic reprogramming is a hallmark of cancer. Cancer cells can reprogram metabolism to support itself proliferation and progression by providing sufficient ATP generation and building blocks.^[^ ^48^ ^]^ Accumulating evidence indicates that glycolysis, lipid metabolism, amino, and glutaminolysis could be regulated by some signaling pathways involved in cancer cell growth and proliferation, eventually resulting in tumor initiation, growth, and metastasis.^[^ ^49^, ^50^ ^]^ Recently, the metabolisms of CSCs, were investigated. For example, CSCs utilize glutamine metabolism for the biosynthesis of lipids, nucleotides, and amino acids. CSCs also depend on lipid metabolism to satisfy their energetic requirements.^[^ ^51^ ^]^ Cholesterol synthesis is involved in cancer cell migration and invasion.^[^ ^52^ ^]^ Recently, cholesterol was reported to enhance the self‐renewal of CSC in colorectal cancer.^[^ ^17^ ^]^ Increased cholesterol synthesis is also important for BCSC propagation and correlates with patient outcome.^[^ ^18^ ^]^ However, there is no detail on cholesterol synthesis. In this study, we unravel that lnc030/SQLE axis could increase cellular cholesterol synthesis to maintain BCSCs stemness through PI3K/Akt signaling cascade. The crosslink between the metabolite cholesterol and BCSCs opens a new direction for the treatment of breast cancer.

In summary, we highlight a novel lnc030 and its functions in BCSCs. Lnc030 can interact with PCBP2 to increase SQLE mRNA stability, a pivotal enzyme in cholesterol synthesis. The enhanced cholesterol involves in the maintenance of BCSCs stemness and progression of breast tumor by stimulating activation of PI3K/Akt signaling. Our findings open new insights for breast cancer treatment and prognosis.

## 4 Experimental Section

##### Cell Culture and Reagents

Human breast cancer cell lines BT549, Hs578T, and MCF7 were cultured in RPMI 1640 medium (Gibco, USA) containing 10% fetal bovine serum (FBS; Gibco, USA) in a 5% CO2 incubator at 37 °C. Cholesterol was purchased from Sigma‐Aldrich, and dissolved in absolute ethanol, at a stock concentration of 25 mM and stored at −20 °C. Actinomycin D (Cayman, USA) was dissolved in DMSO and stored at −20 °C from light.

##### Mammosphere Formation Assay

BCSCs were cultured as described previously.^[^ ^53^ ^]^ Tumor tissues from clinical BC patients were minced and digested with 1 mg mL^−1^ of type 1 collagenase (Sigma) at 37 °C for 30 min to acquire single‐cell suspensions, and used for mammospheres culture. BCSCs were enriched in six‐well plates covered with 2% poly‐2‐hydroxyethyl methacrylate (poly‐HEMA) (Sigma, USA) from BT549, Hs578T, and MCF7 cells using serum‐free medium. The serum‐free medium was composed of DMEM/Ham Nutrient Mixture F‐12 (1:1) (Gibco) with the addition of epidermal growth factor (EGF, 20 ng mL^−1^), human fibroblast growth factor basic (hFGFb, 10 ng mL^−1^), insulin (500 µg mL^−1^) and 2% B27 (Invitrogen) for 14–21 days. BCSCs were kept in culture and passaged every 7 days. The percentage (%) of BCSC sphere formation was calculated as previously described.^[^ ^40^ ^]^ The average volume of BCSC spheres (*N* = 30 spheres) was calculated.

##### Clinical Breast Tumor Samples

Human breast cancer tissues and their adjacent normal mammary tissues (at least 5 cm away from a tumor) were obtained from patients with breast cancer without previous radiotherapy or chemotherapy at the First Affiliated Hospital of Chongqing Medical University. All patients have been informed and consented to involve in this study. And the experiments were approved by the Ethics Committee of Chongqing Medical University (2017‐080).

##### Cell transfection, lentivirus infection, and plasmids constructs

The shRNAs specifically against Lnc030, SQLE, PCBP2, and control hairpins were cloned into PGLVH1/GFP vector; cells were transfected with lentiviral expressing shRNA or shNC for 24 ho. The transfected cells were established by selection with G418 for two weeks. The sequences of the effective shRNAs were provided as follows in Table S2, Supporting Information. The lentiviral of lnc030, SQLE, and PCBP2 overexpression and lentiviral control vector transfected cells for 24 h, then selected with puromycin for two weeks. All lentivirus were acquired from GenePharma (Shanghai, China).

PCBP2 and its three deletion mutants (T1, T2, and T3) were amplified by PCR and inserted into pcDNA3.1‐Flag tagged plasmid to get pcDNA3.1‐Flag‐PCBP2 (FL, full length), pcDNA3.1‐Flag‐PCBP2‐KH1 domain (T1), pcDNA3.1‐Flag‐PCBP2‐KH2 domain (T2), and pcDNA3.1‐Flag‐PCBP2‐KH3 domain (T3) construct. The CDS (coding sequence), 3‐’UTR, and 5’‐UTR of SQLE were amplified and then cloned into pcDNA3.1 vector with biotin RNA labeling.

##### Reverse‐Transcriptional Quantitative PCR (qRT‐PCR)

Total RNA was extracted from secondary generation of mammospheres using Trizol (Takara, Japan) according to the manufacturer's instructions. For reverse transcription of RNA, 1 µg of total RNA was reverse‐transcribed using PrimeScript RT Reagents Kit (Takara, Japan). Quantitative PCR was performed using SYBR premix Ex Taq II Kit (Takara, Japan). *β*‐actin served as the internal control for normalization. Primer sequences for the detected genes were listed in Table S2, Supporting Information.

##### Western Blotting

Indicated cells were lysed in RIPA lysis buffer (Boster, China) with protease inhibitors. The lysates isolated from secondary generation of mammospheres or tumor tissues were separated by 10% SDS‐PAGE and immunoblotted with appropriate antibodies. The specific primary antibodies used in this study were as follows: SQLE (BS71537, Bioworld, 1:500), KLF4 (11880‐1‐AP, Proteintech, 1:500), c‐Myc (10828‐1‐AP, Proteintech, 1:1000), Sox2 (ab97959, Abcam, 1:500), PCBP2 (ab95942, Abcam, 1:1000), Akt (9272, CST, 1:1000), p‐Akt (12 694, CST, 1:1000), PI3K (4255, CST, 1:1000), p‐PI3K (11 508, Signalway Antibody, 1:500), incubated at 4 °C overnight. The membranes were then incubated with the appropriate horseradish peroxidase‐conjugated secondary antibody for 1.5 h. The proteins were visualized by the ECL detection reagents (Bio‐rad, America) on the enhanced chemiluminescence system (Amersham Pharmacia Biotech).

##### Immunohistochemistry (IHC) and Immunofluorescence Staining (IF)

The paraffin‐embedded tumor samples from patients or mice were sectioned into 4 µm thickness, then deparaffinied in xylene, dehydrated, antigen retrieved blocked in 10% goat serum. The tissue sections were incubated with CD44 and CD24 primary antibodies (proteintech, 1:200), SQLE (Bioworld, 1:100) overnight at 4 °C and subsequently followed by HRP secondary antibody for 30 min, then stained with diaminobenizidine. IF staining was carried out following the protocol as described previously.^[^ ^54^ ^]^ The specific antibodies against CD44 (Proteintech, 1:200), c‐Myc (Proteintech, 1:100), SQLE (Bioworld, 1:100) were used. Normal rabbit or Mouse IgG served as a negative control. The results of IHC and IF were visualized and imaged under a Nikon Eclipse 80i microscope (Tokyo, Japan).

##### Immunofluorescence Staining and Fluorescence In Situ Hybridization (FISH)

LncRNA FISH Probe Mix and Fluorescence in situ Hybridization Kit (RIBO Bio, China) was used for detection of the distribution of lnc030 in BT549, Hs578T, and MCF7 cells. FISH was performed according to the manufacturer's protocols. Briefly, the cells were fixed by 4% polyformaldehyde for 15 min and were permeated with 0.5% Triton X‐100 for 30 min. Then, the cells were incubated with lnc030 FISH Probe or control overnight at 4 °C, followed by secondary Cy3‐labeled anti‐rabbit IgG. The nucleus was stained by DIPA. The pictures were accessed by confocal microscope.

##### Flow Cytometry

The secondary generation of spheres was detached by 0.25% trypsin and re‐suspended into single cell in PBS, and then incubated with APC anti‐CD44 (BD) and PE anti‐CD24 (BD) for 30 min at 4 °C protected from light. Then cells were washed three times with PBS and suspended in 500 µl of PBS. The CD44^+^/CD24^−/low^ cells were evaluated by flow cytometry analysis.

##### Cytosolic/Nuclear Fractionation

Separation of nuclear and cytoplasmic RNA was performed with PARI^TM^ Kit (Invitrogen, USA) according to the manufacturer's instructions. The *β*‐actin mRNA was used as cytoplasmic control and U6 RNA as nuclear control. The nuclear and cytoplasmic RNA was further determined by qRT‐PCR. Cellular fractionation assay was verified in BT549, Hs578T, and MCF7 cells.

##### Colony‐Formation Assay

After transfection, the engineered breast cancer cells with lnc030‐knocked down and control cells (1 × 10^3^ well^−1^) were seed into 6‐well plates and kept in culture for 14 days to get cell colonies. After fixed with methanol for 10 min, the colonies were stained with crystal violet for 10 min. Colonies were photographed by a digital camera and colonies were counted.

##### Total Cholesterol Concentrations and ALDH Activity Assay

BCSCs (1 × 10^6^) or breast cancer tissues (2 mg) were harvested. Total cholesterol concentrations were evaluated by Cholesterol/Cholesterol Ester Quantification Kit (ab65359, Abcam) according to the manufacturer's instructions. ALDH activity of breast cancer tissues (50 mg) was detected with the Aldehyde Dehydrogenase Activity Colorimetric Assay Kit (Sigma, MAK082) following the manufacturer's protocol. Experiments were performed three times.

##### RNA Immunoprecipitation (RIP) and RNA Pull‐Down

RIP assays were performed using the Magna RIP RNA‐binding protein immunoprecipitation kit (Millipore, USA) following the manufacturer's instructions. Briefly, 2 × 10^7^ cells were lysed in RIP lysis buffer on ice. Then 5 µg of antibody of interest (PCBP2) was added to incubate with the re‐suspended beads for 30 min at room temperature. The RIP lysis was incubated with beads‐antibody complex in RIP immunoprecipitation buffer at 4 °C overnight. The beads‐bound immunoprecipitate was eluted with elution buffer at 55 °C for 30 min. RNAs were isolated with phenol/chloroform/isoamyl alcohol. Purified RNAs were subjected to qRT‐PCR or reverse transcription PCR.

For antisense oligomer affinity pull‐down assay, 1 µg of sense or antisense biotin‐labeled DNA oligomers against lnc030 or SQLE mRNA were incubated with BT549 spheres lysates for 2 h, respectively. Streptavidin‐coupled agarose beads (Invitrogen) were added to the reaction mix to isolate the RNA‐RNA or RNA‐protein complex, followed by qRT‐PCR and Western blotting.

Lnc030 full‐length sense and antisense were transcribed in vitro with biotin RNA labeling mix and T7 RNA polymerase (Roche, USA) according to the manufacturer's instructions. Biotin‐labeled lnc030 was incubated with total cell lysates of BT549 spheres. The RNA‐protein complex was isolated from magnetic beads using Biotin Elution Buffer. Then the retrieved protein was purified and analyzed by silver staining, followed by Western blotting or mass spectra.

##### Xenograft Mouse Model

To evaluate the tumor formation ability of lnc030, in vivo limiting dilution was performed. 4‐week‐old female nude mice were randomly divided into five groups (*n* = 5 per group). For tumor initiation assay, a series of 1 × 10^3^, 1 × 10^4^, 1 × 10^5^ of MCF‐7 BCSCs (mammosphere cells), lnc030‐knockdown mammospheres with standard chow, lnc030‐knockdown mammospheres with high‐fat/cholesterol diet (HFD), lnc030‐overexpressing mammospheres and control mammospheres were subcutaneously inoculated into mammary fat pads of nude mice respectively. The tumor‐initiation frequency was calculated. For the tumor growth experiment, 2 × 10^6^ BT549 cells in 100 µl PBS were injected into with mammary fat pads of nude mice which divided into eight groups (shNC control, lnc030‐knockdown, SQLE‐knockdown, lnc030‐knockdown with HFD, SQLE‐knockdown with HFD, lnc030 overexpression, lnc030 overexpression + shSQLE, and vector control group). Tumor volumes were determined by caliper measurement (*V* = length × width^2^ × 0.5). At day 49, all mice were sacrificed and tumors were isolated and measured. The acquired tumor tissues were subjected to qRT‐PCR, Western blotting, or immunohistochemistry staining to detect associated gene or protein expression. All mouse experiments were conducted in accordance with standard operating procedures according to the recommendations in the Guide for the Care and Use of Laboratory Animals of Chongqing Medical University and approved by the Ethics Committee of Chongqing Medical University (2017‐081).

##### Statistical Analysis

Statistical analysis was conducted using SPSS 19.0 software. The results are shown as means ± SD from at least three independent experiments. Single comparison between two groups was analyzed by student's *t* test. Comparisons between multiple groups were determined using ANOVA followed by the Student‐Newman‐Keuls multiple‐comparison test. Pearson's correlation analysis was used to evaluate the correlation between two variables. A *p* value <0.05 was considered statistically significant.

## Conflict of Interest

The authors declare no conflict of interest.

## Author Contribution

Y.Q. and M.L. designed the experiments, wrote the manuscript. Y.Q., S.L., P.Z., X.W., M.Z., and M.P. performed the experiments. Y.Q., Y.H., and H.Z. analyzed the data. Q.L., Y.Q. and T.J. collected patient samples. X.C., and ML revised this manuscript.

1

F.
Bray
, 
J.
Ferlay
, 
I.
Soerjomataram
, 
R. L.
Siegel
, 
L. A.
Torre
, 
A.
Jemal
, CA‐Cancer J. Clin.
2018, 68, 394.3020759310.3322/caac.214922

S.
Fan
, 
Z.
Yang
, 
Z.
Ke
, 
K.
Huang
, 
N.
Liu
, 
X.
Fang
, 
K.
Wang
, Biomed. Pharmacother.
2017, 95, 1636.2895066410.1016/j.biopha.2017.09.0763

J.
Kozłowski
, 
A.
Kozłowska
, 
J.
Kocki
, Postepy Hig. Med. Dosw.
2015, 69, 447.10.5604/17322693.1148710258971054

A. M.
Schmitt
, 
H. Y.
Chang
, Cancer Cell
2016, 29, 452.2707070010.1016/j.ccell.2016.03.010PMC48311385

F. M.
Fazal
, 
H. Y.
Chang
, Mol. Cell
2016, 64, 1.2771647910.1016/j.molcel.2016.09.0306

W.
Lin
, 
Q.
Zhou
, 
C. Q.
Wang
, 
L.
Zhu
, 
C.
Bi
, 
S.
Zhang
, 
X.
Wang
, 
H.
Jin
, Int. J. Biol. Sci.
2020, 16, 1194.3217479410.7150/ijbs.40769PMC70533197

Y. P.
Li
, 
F. F.
Duan
, 
Y. T.
Zhao
, 
K. L.
Gu
, 
L. Q.
Liao
, 
H. B.
Su
, 
J.
Hao
, 
K.
Zhang
, 
N.
Yang
, 
Y.
Wang
, Nat. Commun.
2019, 10, 1368.3091100610.1038/s41467-019-08911-wPMC64339528

G.
Yang
, 
X.
Lu
, 
L.
Yuan
, Biochim. Biophys. Acta
2014, 1839, 1097.2515966310.1016/j.bbagrm.2014.08.0129

F.
Peng
, 
T. T.
Li
, 
K. L.
Wang
, 
G. Q.
Xiao
, 
J. H.
Wang
, 
H. D.
Zhao
, 
Z. J.
Kang
, 
W. J.
Fan
, 
L. L.
Zhu
, 
M.
Li
, 
B.
Cui
, 
F. M.
Zheng
, 
H. J.
Wang
, 
E. W.
Lam
, 
B.
Wang
, 
J.
Xu
, 
Q.
Liu
, Cell Death Dis.
8, e2569 (2018).10.1038/cddis.2016.438PMC53863572810284510

M.
Al‐Hajj
, 
M. S.
Wicha
, 
A.
Benito‐Hernandez
, 
S. J.
Morrison
, 
M. F.
Clarke
, Proc. Natl. Acad. Sci. U.S.A.
2003, 100, 3983.1262921810.1073/pnas.0530291100PMC15303411

E.
Charafe‐Jauffret
, 
C.
Ginestier
, 
F.
Bertucci
, 
O.
Cabaud
, 
J.
Wicinski
, 
P.
Finetti
, 
E.
Josselin
, 
J.
Adelaide
, 
T. T.
Nguyen
, 
F.
Monville
, 
J.
Jacquemier
, 
J.
Thomassin‐Piana
, 
G.
Pinna
, 
A.
Jalaguier
, 
E.
Lambaudie
, 
G.
Houvenaeghel
, 
L.
Xerri
, 
A.
Harel‐Bellan
, 
M.
Chaffanet
, 
P.
Viens
, 
D.
Birnbaum
, Cancer Res.
2013, 73, 7290.2414234410.1158/0008-5472.CAN-12-470412

M.
Shaimerdenova
, 
O.
Karapina
, 
D.
Mektepbayeva
, 
K.
Alibek
, 
D.
Akilbekova
, Infect. Agent Cancer
2017, 12, 18.2834464010.1186/s13027-017-0128-7PMC536457213

G.
Baniebrahimi
, 
F.
Mir
, 
R.
Khanmohammadi
, Cancer Cell Int.
2020, 20, 113.3228030510.1186/s12935-020-01192-0PMC713742114

X.
Ding
, 
W.
Zhang
, 
S.
Li
, 
H.
Yang
, Am. J. Cancer Res.
2019, 9, 219.30906624PMC640598115

G. D.
Batty
, 
M.
Kivimäki
, 
R.
Clarke
, 
G.
Davey Smith
, 
M. J.
Shipley
, Cancer Causes Control
2011, 22, 311.2111684310.1007/s10552-010-9691-6PMC322694916

J.
Zhao
, 
X.
Zhang
, 
T.
Gao
, 
S.
Wang
, 
Y.
Hou
, 
P.
Yuan
, 
Y.
Yang
, 
T.
Yang
, 
J.
Xing
, 
J.
Li
, 
S.
Liu
, Cell Death Dis.
2020, 11, 25.3193258110.1038/s41419-019-2221-xPMC695752417

C.
Wang
, 
P.
Li
, 
J.
Xuan
, 
C.
Zhu
, 
J.
Liu
, 
L.
Shan
, 
Q.
Du
, 
Y.
Ren
, 
J.
Ye
, Cell. Physiol. Biochem.
2017, 42, 729.2861841710.1159/00047789018

S.
Ehmsen
, 
M. H.
Pedersen
, 
G.
Wang
, 
M. G.
Terp
, 
A.
Arslanagic
, 
B. L.
Hood
, 
T. P.
Conrads
, 
R.
Leth‐Larsen
, 
H. J.
Ditzel
, Cell Rep.
2019, 27, 3927.3124242410.1016/j.celrep.2019.05.10419

S. A.
Mani
, 
W.
Guo
, 
M. J.
Liao
, 
E. N.
Eaton
, 
A.
Ayyanan
, 
A. Y.
Zhou
, 
M.
Brooks
, 
F.
Reinhard
, 
C. C.
Zhang
, 
M.
Shipitsin
, 
L. L.
Campbell
, 
K.
Polyak
, 
C.
Brisken
, 
J.
Yang
, 
R. A.
Weinberg
, Cell
2008, 133, 704.1848587710.1016/j.cell.2008.03.027PMC272803220

J. L.
Rinn
, 
H. Y.
Chang
, Annu. Rev. Biochem.
2012, 81, 145.2266307810.1146/annurev-biochem-051410-092902PMC385839721

M. W.
Helms
, 
D.
Kemming
, 
H.
Pospisil
, 
U.
Vogt
, 
H.
Buerger
, 
E.
Korsching
, 
C.
Liedtke
, 
C. M.
Schlotter
, 
A.
Wang
, 
S. Y.
Chan
, 
B. H.
Brandt
, Br. J. Cancer
2008, 99, 774.1872866810.1038/sj.bjc.6604556PMC252813722

T. Z.
Parris
, 
A.
Kovács
, 
S.
Hajizadeh
, 
S.
Nemes
, 
M.
Semaan
, 
M.
Levin
, 
P.
Karlsson
, 
K.
Helou
, Oncogenesis
2014, 3, e95.2466292410.1038/oncsis.2014.8PMC403838923

Y.
Wang
, 
W.
Chen
, 
J.
Lian
, 
H.
Zhang
, 
B.
Yu
, Cell Death Differ.
2020, 27, 695.3132074910.1038/s41418-019-0381-yPMC720608424

Z.
Xin
, 
W.
Han
, 
Z.
Zhao
, 
Q.
Xia
, 
B.
Yin
, 
J.
Yuan
, 
X.
Peng
, PLoS One
2011, 6, e25419.2202239110.1371/journal.pone.0025419PMC319114925

Q.
Gu
, 
X.
Yang
, Science
2019, 363, 1085.3070515310.1126/science.aav1749PMC646935426

E. S.
Mathews
, 
B.
Appel
, J. Neurosci.
2016, 36, 7628.2744514110.1523/JNEUROSCI.0726-16.2016PMC495157327

J. K.
Jeong
, 
J. W.
Seol
, 
M. H.
Moon
, 
J. S.
Seo
, 
Y. J.
Lee
, 
J. S.
Kim
, 
S. Y.
Park
, Biochem. Biophys. Res. Commun.
2010, 401, 516.2087540010.1016/j.bbrc.2010.09.07828

A. H.
Reis
, 
M. M.
Moreno
, 
L. A.
Maia
, 
F. P.
Oliveira
, 
A. S.
Santos
, 
J. G.
Abreu
, Mech. Dev.
2016, 142, 30.2768754110.1016/j.mod.2016.09.00129

S.
Chatterjee
, 
P. C.
Sil
, Pharmacol. Res.
2019, 142, 251.3082645610.1016/j.phrs.2019.02.02730

L.
Yang
, 
P.
Shi
, 
G.
Zhao
, 
J.
Xu
, 
W.
Peng
, 
J.
Zhang
, 
G.
Zhang
, 
X.
Wang
, 
Z.
Dong
, 
F.
Chen
, 
H.
Cui
, Signal Transduction Targeted Ther.
2020, 5, 8.10.1038/s41392-020-0110-5PMC70052973229603031

S.
He
, 
S.
Yang
, 
Y.
Zhang
, 
X.
Li
, 
D.
Gao
, 
Y.
Zhong
, 
L.
Cao
, 
H.
Ma
, 
Y.
Liu
, 
G.
Li
, 
S.
Peng
, 
C.
Shuai
, Cell Death Dis.
2019, 10, 947.3182707610.1038/s41419-019-2148-2PMC690639332

Q.
Zhou
, 
J.
Guo
, 
W.
Huang
, 
X.
Yu
, 
C.
Xu
, 
X.
Long
, Mol. Oncol.
2020, 14, 2231.3233599810.1002/1878-0261.12700PMC746337133

H.
Zhang
, 
P.
Wei
, 
W.
Lv
, 
X.
Han
, 
J.
Yang
, 
S.
Qin
, Cell Biosci.
2019, 9, 81.3159211410.1186/s13578-019-0345-4PMC677566734

J. T.
Hua
, 
S.
Chen
, 
H. H.
He
, Trends Genet.
2019, 35, 840.3162387210.1016/j.tig.2019.08.00435

K. T.
Jin
, 
J. Y.
Yao
, 
X. L.
Fang
, 
H.
Di
, 
Y. Y.
Ma
, Life Sci.
2020, 252, 117647.3227593510.1016/j.lfs.2020.11764736

F.
Crudele
, 
N.
Bianchi
, 
E.
Reali
, 
M.
Galasso
, 
C.
Agnoletto
, 
S.
Volinia
, Mol. Cancer
2020, 19, 61.3218847210.1186/s12943-020-01181-xPMC707943337

Y. P.
Li
, 
Y.
Wang
, J. Genet. Genomics
2015, 42, 99.2581908610.1016/j.jgg.2015.02.00238

Y.
Wang
, 
L.
He
, 
Y.
Du
, 
P.
Zhu
, 
G.
Huang
, 
J.
Luo
, 
X.
Yan
, 
B.
Ye
, 
C.
Li
, 
P.
Xia
, 
G.
Zhang
, 
Y.
Tian
, 
R.
Chen
, 
Z.
Fan
, Cell Stem Cell
2015, 16, 413.2584297910.1016/j.stem.2015.03.00339

W.
Li
, 
L.
Zhang
, 
B.
Guo
, 
J.
Deng
, 
S.
Wu
, 
F.
Li
, 
Y.
Wang
, 
J.
Lu
, 
Y.
Zhou
, Mol. Cancer
2019, 18, 22.3073686010.1186/s12943-019-0949-7PMC636780940

M.
Zhou
, 
Y.
Hou
, 
G.
Yang
, 
H.
Zhang
, 
G.
Tu
, 
Y. E.
Du
, 
S.
Wen
, 
L.
Xu
, 
X.
Tang
, 
S.
Tang
, 
L.
Yang
, 
X.
Cui
, 
M.
Liu
, Stem Cells
2016, 34, 55.2641836510.1002/stem.2219PMC483213741

I.
Gonzalez‐Moro
, 
A.
Olazagoitia‐Garmendia
, Proc. Natl. Acad. Sci. U.S.A.
2020, 117, 9022.3228440410.1073/pnas.1914353117PMC718322142

A.
Zhang
, 
J. C.
Zhao
, 
J.
Kim
, 
K. W.
Fong
, 
Y. A.
Yang
, 
D.
Chakravarti
, 
Y. Y.
Mo
, 
J.
Yu
, Cell Rep.
2015, 13, 209.2641168910.1016/j.celrep.2015.08.069PMC475746943

G.
Cirmena
, 
P.
Franceschelli
, 
E.
Isnaldi
, 
L.
Ferrando
, 
M.
De Mariano
, 
A.
Ballestrero
, 
G.
Zoppoli
, Cancer Lett.
2018, 425, 13.2959688810.1016/j.canlet.2018.03.03444

D. N.
Brown
, 
I.
Caffa
, 
G.
Cirmena
, 
D.
Piras
, 
A.
Garuti
, 
M.
Gallo
, 
S.
Alberti
, 
A.
Nencioni
, 
A.
Ballestrero
, 
G.
Zoppoli
, Sci. Rep.
2016, 6, 19435.2677706510.1038/srep19435PMC472602545

D.
Liu
, 
C. C.
Wong
, 
L.
Fu
, Sci. Transl. Med.
2018, 10, eaap9840.2966985510.1126/scitranslmed.aap984046

M.
Polycarpou‐Schwarz
, 
M.
Groß
, 
P.
Mestdagh
, 
J.
Schott
, 
S. E.
Grund
, 
C.
Hildenbrand
, 
J.
Rom
, 
S.
Aulmann
, 
H. P.
Sinn
, 
J.
Vandesompele
, Oncogene.
2018, 37, 4750.2976515410.1038/s41388-018-0281-547

Y.
Qin
, 
Y.
Zhang
, 
Q.
Tang
, 
L.
Jin
, 
Y.
Chen
, Acta Biochim. Biophys. Sin.
2017, 49, 138.2806958610.1093/abbs/gmw12748

S.
McGuirk
, 
Y.
Audet‐Delage
, 
J.
St‐Pierre
, Trends Cancer
2020, 6, 49.3195278110.1016/j.trecan.2019.11.00949

J.
Serpa
, Adv. Exp. Med. Biol.
2020, 1219, 1.3213069110.1007/978-3-030-34025-4_150

M. K. L.
Fung
, 
G. C.
Chan
, J. Hematol. Oncol.
2017, 10, 144.2875068110.1186/s13045-017-0509-9PMC553096251

S. Y.
Lee
, 
M. K.
Ju
, Oxid Med Cell Longev.
2018, 2018, 1027453.3067116810.1155/2018/1027453PMC632353352

L.
Zhang
, 
S. B.
Kim
, 
K.
Luitel
, 
J. W.
Shay
, Mol. Cancer Ther.
2018, 17, 943.2946727310.1158/1535-7163.MCT-17-088753

D.
Ponti
, 
A.
Costa
, 
N.
Zaffaroni
, 
G.
Pratesi
, 
G.
Petrangolini
, 
D.
Coradini
, 
S.
Pilotti
, 
M. A.
Pierotti
, 
M. G.
Daidone
, Cancer Res.
2005, 65, 5506.1599492010.1158/0008-5472.CAN-05-062654

F.
Ma
, 
X.
Liu
, 
S.
Zhou
, 
W.
Li
, 
C.
Liu
, 
M.
Chadwick
, 
C.
Qian
, Cancer Lett.
2019, 450, 63.3077142510.1016/j.canlet.2019.02.008

## Supporting information

Supporting Information
