# Supplementary material for: A Novel Long Non‐Coding RNA lnc030 Maintains Breast Cancer Stem Cell Stemness by Stabilizing SQLE mRNA and Increasing Cholesterol Synthesis
Source: Adv Sci (Weinh). 2020 Nov 30;8(2):2002232. doi: 10.1002/advs.202002232 (PMC7816696; doi:10.1002/advs.202002232)
Supplement: Supplementary file 1 — Supporting Information [file ADVS-8-2002232-s001.pdf]

© 2020 Wiley-VCH GmbH

ADVANCED SCIENCE
Open Access

Supporting Information

for *Adv. Sci.,* DOI: 10.1002/advs.202002232

A novel long non-coding RNA lnc030 maintains breast cancer stem cell stemness by stabilizing SQLE mRNA and increasing cholesterol synthesis

Yilu Qin[a], Yixuan Hou[b], Shuiqing Liu[a], Pengpeng Zhu[a], Xueying Wan[a], Maojia Zhao[a], Meixi Peng[a], Huan Zeng[a], Qiao Li[a], Ting Jin[a], Xiaojiang Cui[c], Manran Liu[a,]

Fig. S1

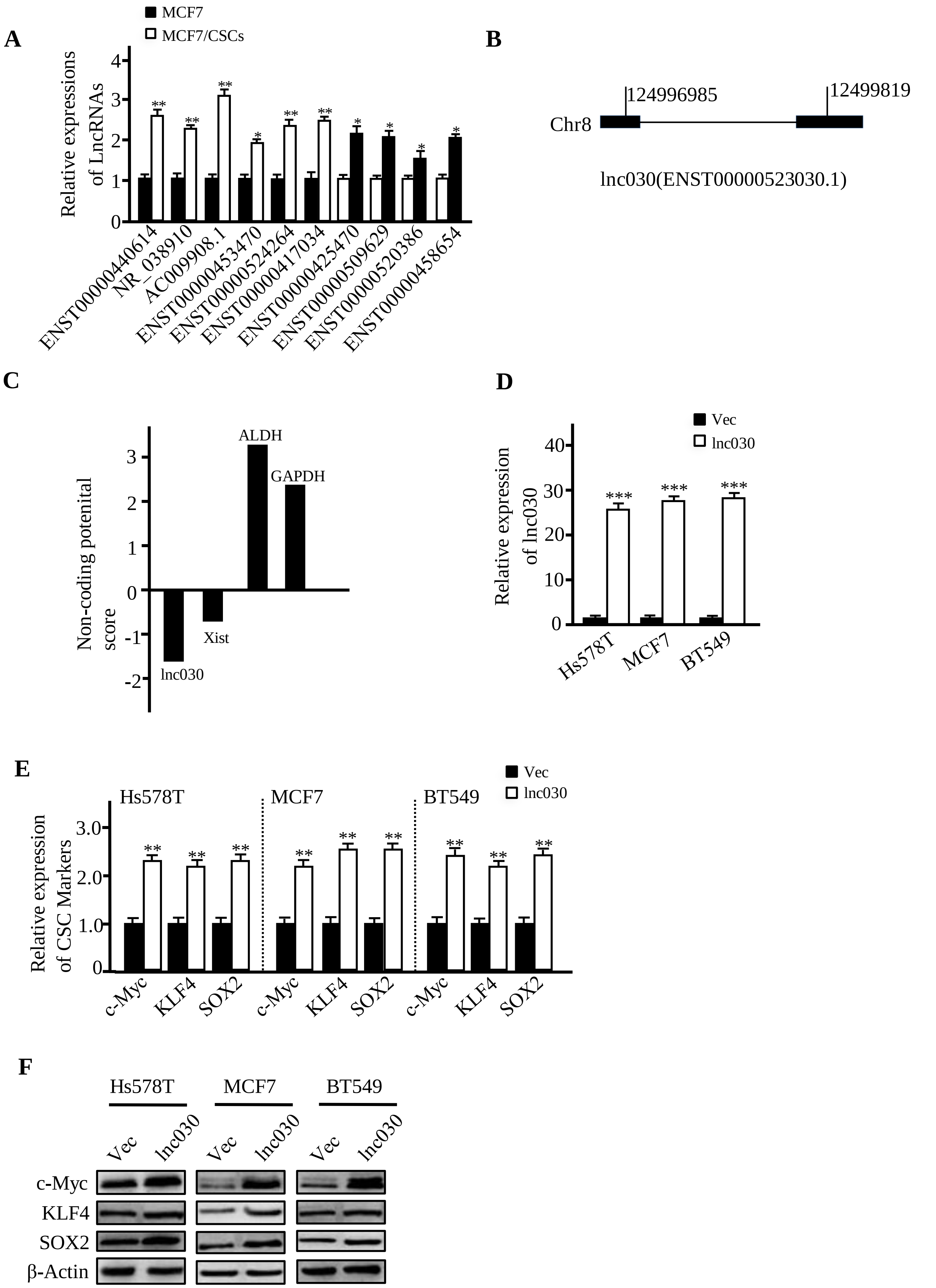

A
MCF7
MCF7/CSCs
Relative expressions of LncRNAs
0
1
2
3
4
ENST00000440614
NR_038910
AC009908.1
ENST00000453470
ENST00000524264
ENST00000417034
ENST00000425470
ENST00000509629
ENST00000520386
ENST00000458654
B
Chr8
124996985
12499819
lnc030(ENST00000523030.1)
C
Non-coding potenital score
ALDH
GAPDH
Xist
lnc030
3
2
1
0
-1
-2
D
Vec
lnc030
Relative expression of lnc030
40
30
20
10
0
Hs578T
MCF7
BT549
E
Vec
lnc030
Hs578T
MCF7
BT549
Relative expression of CSC Markers
3.0
2.0
1.0
0
c-Myc
KLF4
SOX2
F
Hs578T
MCF7
BT549
Vec
lnc030
c-Myc
KLF4
SOX2
β-Actin

Supplementary Figure legends

Figure S1. Lnc030 is highly expressed in BCSCs

(A). The expressions of LncRNAs were detected by qRT-PCR in MCF7 and MCF7/CSCs. qRT-PCR data are shown as means ± SD (*p<0.05, **p<0.01). (B). Annotated map of lnc030 on chromosome 8. (C). Coding potency of Lnc030 sequence was analyzed by Coding Protential Calculation. X inactivation-specific transcript (XIST) served as a control non-coding gene. Glyceraldehyde-3-phosphate dehydrogenase (GAPDH) and β-actin serve as control coding genes. Scores more than 0 suggest a coding potential of lncRNA, whereas scores less than 0 represent no coding potential of the evaluated LncRNA. (D). Lnc030 was transfected into BCs by lentivirus. Lnc030-overexpressing stable cells were established. Data are shown as means ± SD (***p<0.001). (E, F). The expression of pluripotent transcription factors c-Myc, KLF4, and SOX2 was determined in the indicated breast cells with ectopic lnc030 and vector control by qRT-PCR (E) and Western blotting (F). qRT-PCR data are shown as means ± SD (**p<0.01).

Fig. S2

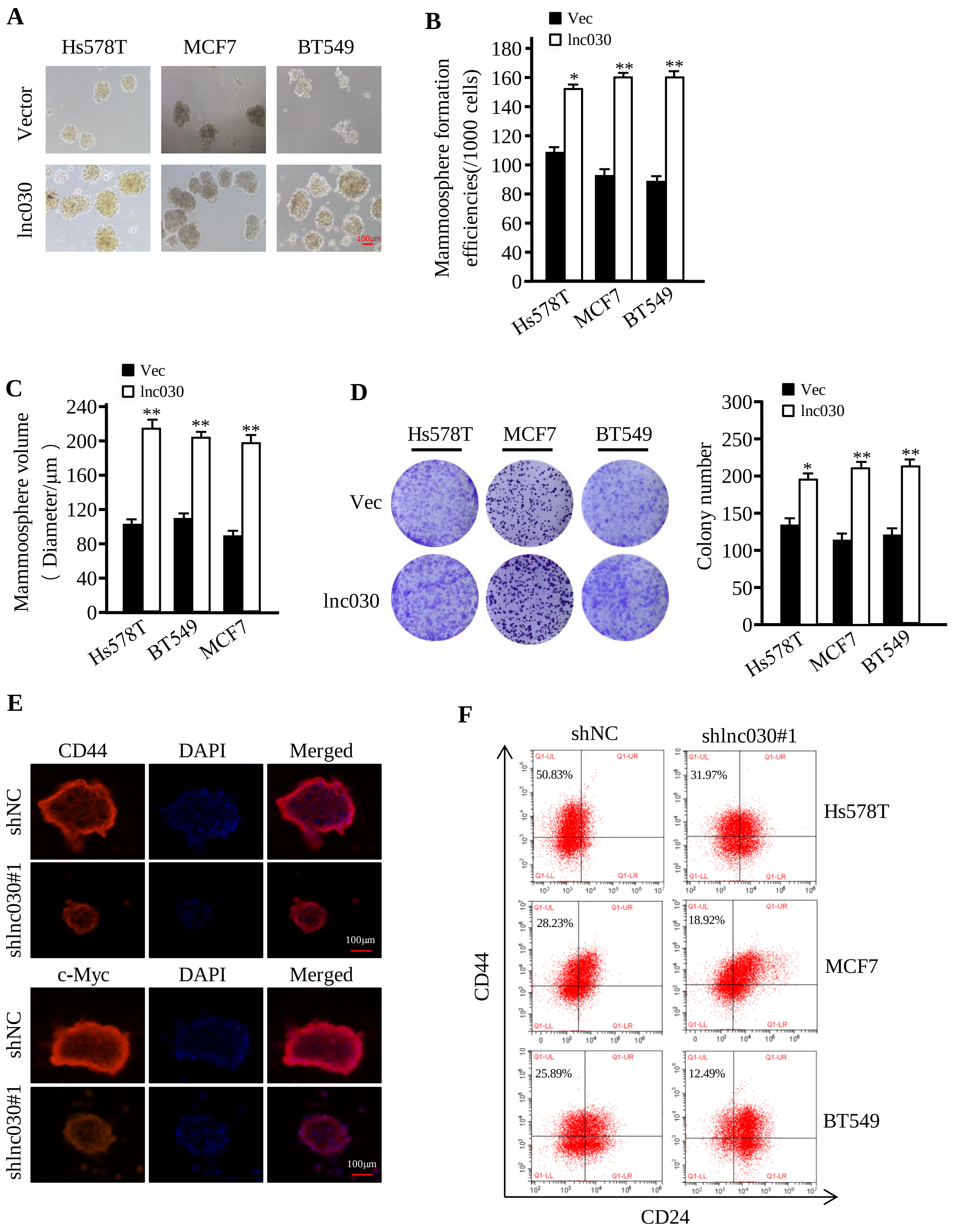

A
Hs578T
MCF7
BT549
Vector
lnc030
100μm
B
Vec
lnc030
Mammosphere formation efficiencies(/1000 cells)
0
20
40
60
80
100
120
140
160
180
*
**
**
Hs578T
MCF7
BT549
C
Vec
lnc030
Mammosphere volume (Diameter/μm)
0
40
80
120
160
200
240
**
**
**
Hs578T
BT549
MCF7
D
Hs578T
MCF7
BT549
Vec
lnc030
Vec
lnc030
Colony number
0
50
100
150
200
250
300
*
**
**
Hs578T
MCF7
BT549
E
CD44
DAPI
Merged
shNC
shlnc030#1
100μm
c-Myc
DAPI
Merged
shNC
shlnc030#1
100μm
F
shNC
shlnc030#1
50.83%
31.97%
Hs578T
28.23%
18.92%
MCF7
25.89%
12.49%
BT549
CD44
CD24

Figure S2. Lnc030 is required for maintaining BCSCs stemness

(A). Ectopic lnc030 increased sphere formation abilities in breast cancer cells. The representative mammopheres are shown (Scale bar, 100μm). (B, C). The sphere-formation efficiencies (left panel) and spheroid sizes (right panel) were quantified for the indicated engineered breast cancer cells and their control cells in (A). The data represent means ± SD (n=3; *p<0.05; **p<0.01). (D). Colonies of the indicated breast cancer cells were stained with crystal violet and photographed after 14 days of incubation. The numbers of colonies were counted and plotted in columns (right panel). The data represent means ± SD (*p<0.05; **p<0.01). (E). Immunofluorescence staining of stemness biomarkers (CD44 and c-MYC) in lnc030-silenced BCSCs and control BCSCs (Scale bar, 100 μm). (F). Flow cytometry was used to determine the percentage of $CD44^{+}/CD24^{-}$ cell population in secondary generation spheres derived from Hs578T, MCF7 and BT549 with shNC or shlnc030.

Fig. S3

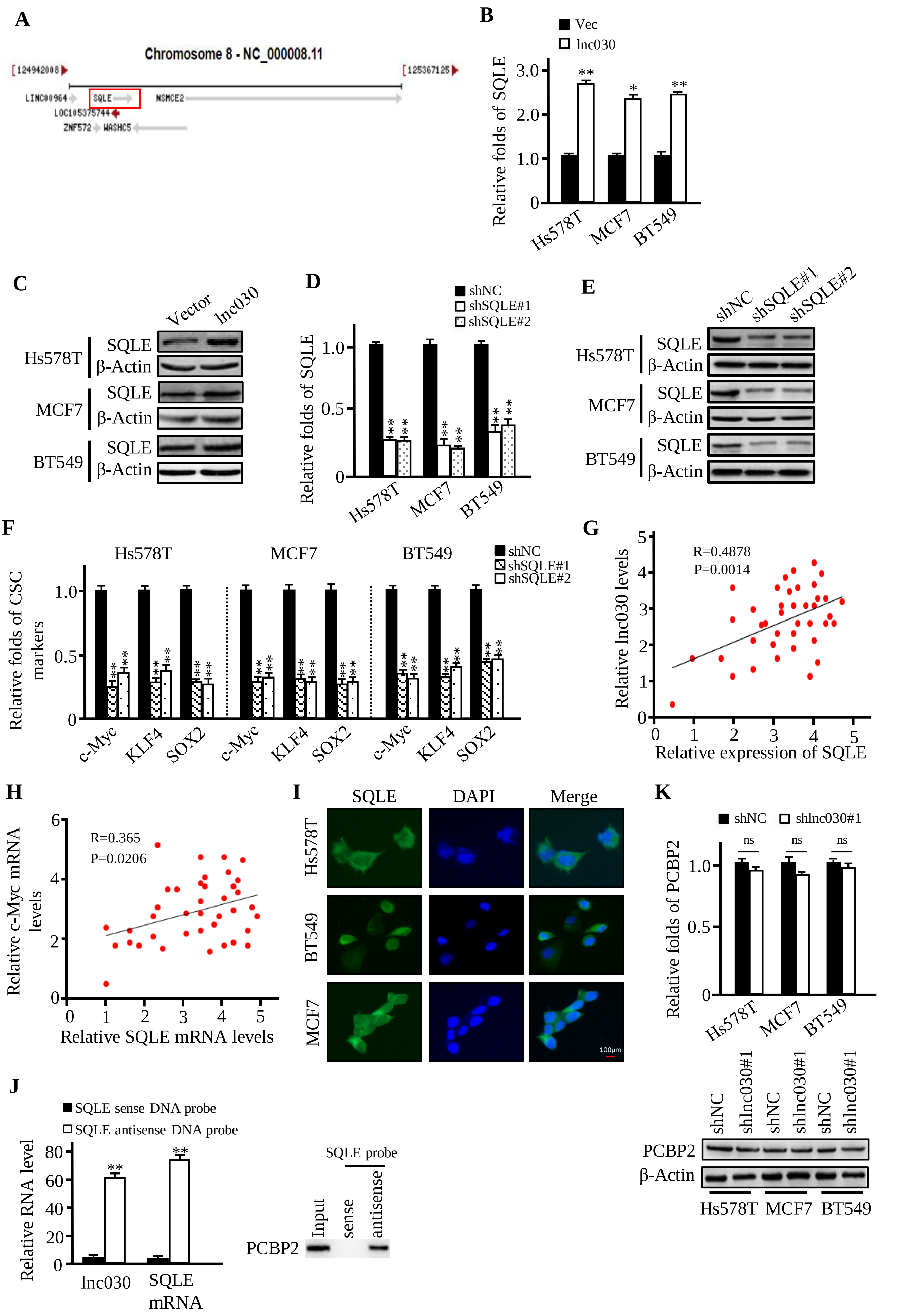

A
Chromosome 8 - NC_000008.11
124942008
125367125
LINC00964
SQLE
NSMCE2
LOC105375744
ZNF572
WASHC5
B
Vec
lnc030
Relative folds of SQLE
Hs578T
MCF7
BT549
C
Vector
lnc030
SQLE
β-Actin
D
shNC
shSQLE#1
shSQLE#2
Relative folds of SQLE
E
F
Relative folds of CSC markers
c-Myc
KLF4
SOX2
G
R=0.4878
P=0.0014
Relative lnc030 levels
Relative expression of SQLE
H
R=0.365
P=0.0206
Relative c-Myc mRNA levels
Relative SQLE mRNA levels
I
SQLE
DAPI
Merge
100μm
J
SQLE sense DNA probe
SQLE antisense DNA probe
Relative RNA level
lnc030
SQLE mRNA
SQLE probe
Input
sense
antisense
PCBP2
K
shNC
shlnc030#1
ns
Relative folds of PCBP2
PCBP2
β-Actin

Figure S3. Lnc030 promotes SQLE expression to maintain BCSCs stemness

(A). The location of lnc030 and SQLE in human chromosome 8. (B, C). mRNA (B) and protein levels (C) of SQLE in lnc030-overexpressing breast cancer cells and control cells were determined by qRT-PCR and Western blotting. The data represent means ± SD (*p<0.05; **p<0.01). (D, E). Knockdown efficiencies of SQLE using two independent shRNAs were evaluated by qRT-PCR (D) and Western blotting (E) in BCSCs. Data are shown as means ± SD (**p<0.01). (F). The mRNA levels of CSC markers c-Myc, Klf4, and Sox2 mRNA levels were detected in SQLE-knockdown and control BCSCs. The data represent means ± SD (**p<0.01). (G, H). The positive correlation between lnc030 and SQLE (G), or c-Myc and SQLE (H) are shown in breast cancer tissues. The data represent means ± SD (n=40). (I). SQLE is mainly located in the cytoplasm of breast cancer cells checked by immunofluorescence (Scale bar, 100 μm). (J). Lysates from BT549 spheres were incubated with either sense or antisense biotin-labeled probe against SQLE mRNA. The pull-down precipitates were analyzed by qRT-PCR or Western blotting to detect the indicated RNAs and protein PCBP2, respectively. Data shown are the mean ± SD (n=3; **p<0.01). (K).The mRNA and protein levels of PCBP2 in spheres form Hs578T, BT549 and MCF7 transfected shNC or shlnc030#1 are shown. Data represent means ± SD (n = 3).

Fig. S4

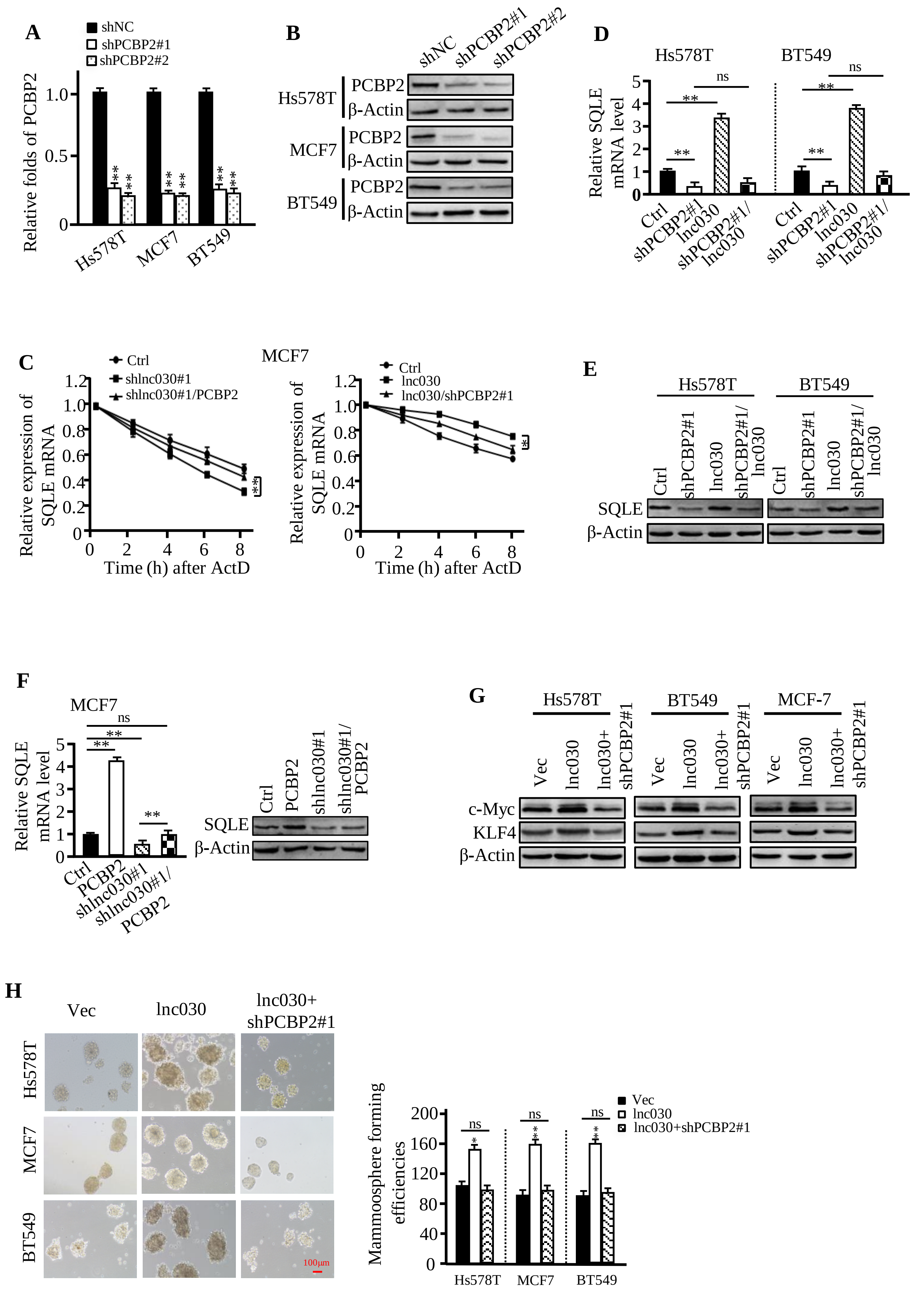

A
shNC
shPCBP2#1
shPCBP2#2
Relative folds of PCBP2
Hs578T
MCF7
BT549
B
shNC
shPCBP2#1
shPCBP2#2
PCBP2
β-Actin
D
Hs578T
BT549
Relative SQLE mRNA level
Ctrl
shPCBP2#1
lnc030
shPCBP2#1/lnc030
ns
C
MCF7
Ctrl
shlnc030#1
shlnc030#1/PCBP2
lnc030
lnc030/shPCBP2#1
Relative expression of SQLE mRNA
Time (h) after ActD
E
SQLE
F
PCBP2
shlnc030#1/PCBP2
G
MCF-7
Vec
lnc030+shPCBP2#1
c-Myc
KLF4
H
100μm
Mammoosphere forming efficiencies

Figure S4. Lnc030 cooperates with PCBP2 to increase SQLE mRNA stability

(A, B). Knockdown efficiencies of PCBP2 using two independent shRNAs were evaluated by qRT-PCR (A) and Western blotting (B) in BCSCs. Data are shown as means ± SD (**p<0.01). (C). The degradation rates of SQLE mRNA in the engineered MCF7 cells and their controls were evaluated by qRT-PCR. The data represent means ± SD (*p<0.05; **p<0.01). (D-F). SQLE mRNA or protein was detected by qRT-PCR and Western blotting in the indicated mammospheres from Hs578T, BT549 cells (D, E), and MCF-7 cells (F) (**p<0.01). (G). Protein levels of CSC markers c-Myc, KLF4 were assessed in BC cells with control vector, lnc030, or lnc030 combined with shPCBP2#1. The data represent means ± SD. (H). The mammosphere-formation efficiency was evaluated for the breast cancer cells infected with control vector, lnc030, or lnc030 combined with shPCBP2#1. The data represent means ± SD (n=3; *p<0.05; **p<0.01, Scale bar, 100 µm).

Fig. S5

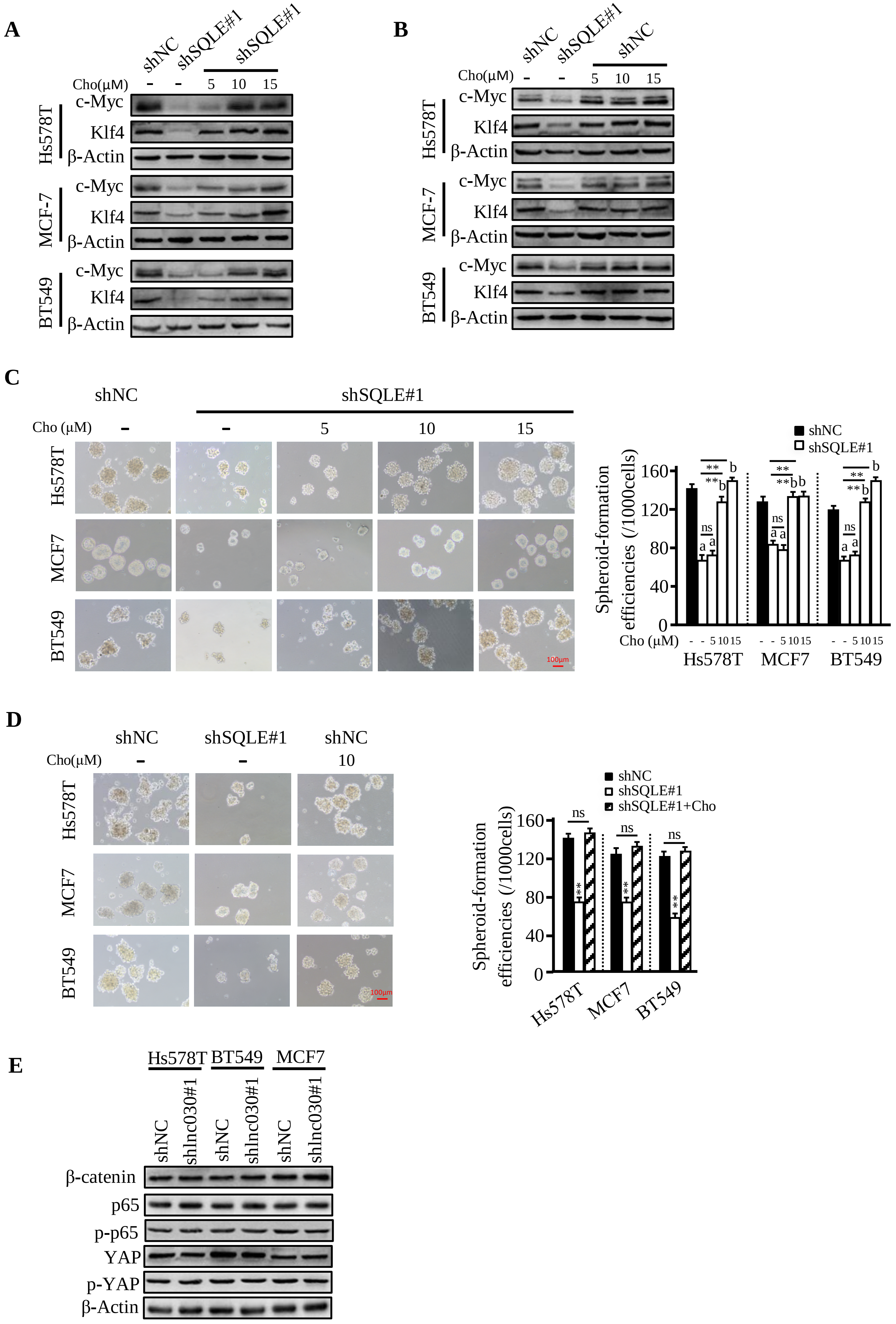
A
shNC
shSQLE#1
shSQLE#1
Cho(μM)
5 10 15
c-Myc
Klf4
β-Actin
Hs578T
MCF-7
BT549
B
shNC
shSQLE#1
shNC
C
shNC
shSQLE#1
Cho (μM)
5 10 15
Hs578T
MCF7
BT549
100μm
shNC
shSQLE#1
Spheroid-formation efficiencies (/1000cells)
160
120
80
40
0
ns
a
b
**
Cho (μM)
- - 5 10 15
D
shNC
shSQLE#1
shNC
Cho(μM)
10
shSQLE#1+Cho
E
Hs578T
BT549
MCF7
shNC
shlnc030#1
β-catenin
p65
p-p65
YAP
p-YAP
β-Actin

Figure S5. Intracellular cholesterol involves in maintenance of BCSC stemness

(A, B). c-Myc and KLF4 proteins were detected by Western blotting in SQLE-knockdown BCSCs (A) and control BCSCs (B) under treatment with or without cholesterol (labeled Cho) at 5μM, 10μM, or 15μM for 48 hours. (C). The sphere-forming capacities were assessed in control BC cells and SQLE-knockdown BC cells treated with cholesterol at 5μM, 10μM or 15μM for every 3 days. Data are shown as means ± SD (a, p<0.01, vs shNC + vehicle; b, p>0.05, vs shNC + vehicle; ns, p>0.05, vs shSQLE#1 + vehicle; **p<0.01, vs shSQLE#1 + vehicle,Scale bar, 100 μm). (D). The spheroid formation abilities were measured in SQLE-knockdown BC cells and control BC cells treated with or without cholesterol at 10μM. Data are shown as means ± SD (**p<0.01, Scale bar, 100 μm). (E). The expressions of β-catenin, p65, p-p65, YAP ,p-YAP were detected by Western blotting in lnc030-knockdown BCSCs and their control BCSC.

Fig. S6

A

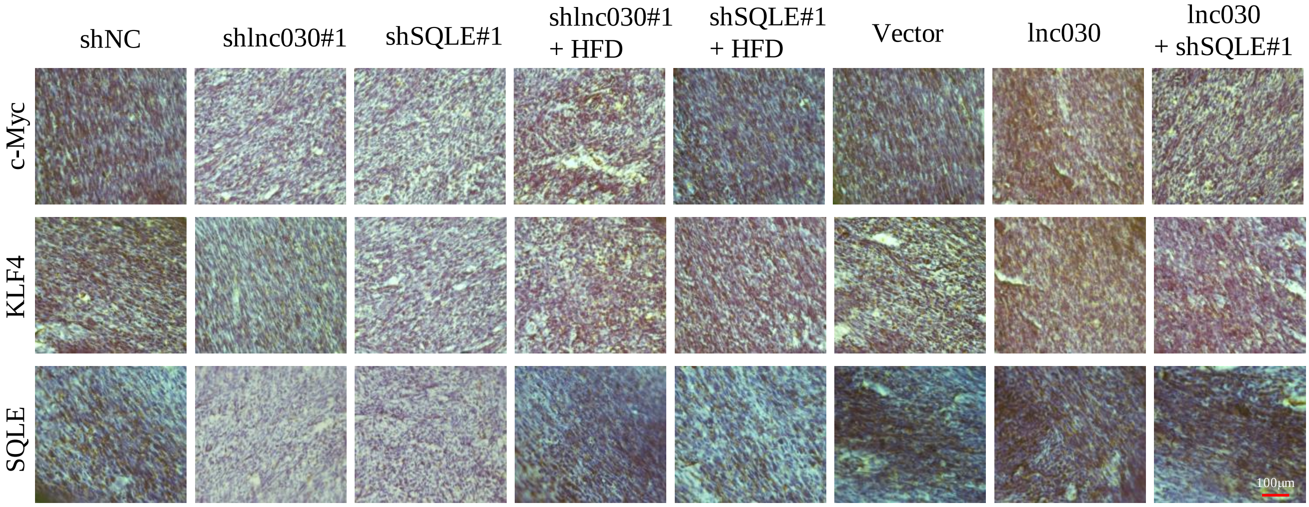
shNC
shlnc030#1
shSQLE#1
shlnc030#1 + HFD
shSQLE#1 + HFD
Vector
lnc030
lnc030 + shSQLE#1
c-Myc
KLF4
SQLE
100μm

Figure S6. Lnc030 Promotes Breast Cancer Initiation and Progression

(A). Representative immunohistochemical staining of SQLE, c-Myc and KLF4 protein expression in tumor of xenografts (Scale bar, 100 μm).

Supplementary Table 1. The correlation between lnc030 expression and clinicopathological features of breast cancer patients (n=40)

**Variables**	**Expression of lnc030**		***p*-value**
	**Low(20)**	**High(20)**	
Age			0.351
<50	12	9	
⩾50	8	11	
Tumor size			0.109
⩽2	10	5	
2-5	7	7	
>5	3	8	
Lymph node metastasis			0.023*
Yes	4	15	
No	16	5	
Distant metastasis			0.018*
Yes	2	17	
No	18	3	
Grade			0.026*
Ⅰ	11	3	
Ⅱ	5	8	
Ⅲ	4	11	

*p<0.05 was considered to be statistically significant.

Supplementary Table 2. Sequences that target specific genes

Gene name	Sequence
lnc030	FW 5'-ACGTTTCCATTCATTCCGCTA-3'
	REV 5'-TCTGATTCCTCTTTCGGACT-3'
c-Myc	FW 5'-AAAGGCCCCCAAGGTAGTTA-3'
	REV 5'-GCACAAGAGTTCCGTAGCTG-3'
KLF4	FW 5'-GAACTGACCAGGCACTACCG-3'
	REV 5'-TTCTGGCAGTGTGGGTCTTA-3'
SOX2	FW 5'-GCACATGAACGGCTGGAGCAACG-3'
	REV 5'-TGCTGCGAGTAGGACATGCTGTA-3'
SQLE	FW 5'-CAGATAATCTCCGCCTTGACC-3'
	REV 5'-AAAGTGATGAAGTCCCCGAAC-3'
PCBP2	FW 5'-GAACACTGCTCGACATGGAC-3'
	REV 5'-R:CTCGCGCATCTTCTTAACTGA-3'
β-actin	FW 5'-TGACGTGGACATCCGCAAAG-3'
	REV 5'-CTGGAAGGTGGACAGCGAGG-3'
shlnc030#1	5'-ATGCTAGCGGAATGAATGGTT-3'
shlnc030#2	5'-TTGTAACATACAGGGCAGGTT-3'
shSQLE#1	5'-GCACCACAGTTTAAAGCAAAT-3'
shSQLE#2	5'-GCTCAGGCTCTTTATGAATTA-3'
shPCBP2#1	5'-CCTGAGAGAATTATCACTT-3'
shPCBP2#2	5'-CCATCATTGAGTGTGTCAATT-3'

Sequences of primers used for pcDNA3.1-lnc030 construction

Full-length	F:GGCGCTCTGGGGCCGGGATC
	R:TGTTTTACCAAATAATTATT
1-150	F:GGCGCTCTGGGGCCGGGATC
	R:ATGGAAACGTTCCGACCCGA
151-300	F:TCATTCCGCTAGCATTTATT
	R:AAAGGATGTTCGAGTTCATG
301-412	F:GCAATTCTGTATCAGATACT
	R:TGTTTTACCAAATAATTATTT

Sequences of primers used for pcDNA3.1-SQLE mRNA construction

5'UTR	F:GAGACGCGTGGAGCCTGGCG
	R:GGTTCCAAGGCTTCTTTGGTG
CDS	F:ATGTGGACTTTTCTGGGCAT
	R:TTAATGAACCATATACTTCAT
3'UTR	F:GCTTAAAGGGGAACCATTTG
	R:TTCATTCTTCATTTTATTTG

Sequences of primers used for pcDNA3.1-PCBP2 construction

Full-length	F:ATGGACACCGGTGTGATTGA
	R:CTAGCTGCTCCCCATGCCAC

1-288	F:ATGGACACCGGTGTGATTGA
	R:GGGTCTACTGGCAGCTGTGC
226-858	F: ATTGACAAACTGGAAGAGGA
	R: TGAGCAGATGCATCCAAACC
487-1095	F: TGCGTGGTCATGTTGGAGAC
	R: GCTGCTCCCCATGCCACCCGT

Primers for in vitro transcription

The T7 RNA polymerase sequence(T7) was 5'taatacgactcactataggg'3

lnc030(sense)	**F:** (T7)GGCGCTCTGGGGCCGGGATCGGCGTCGGGG
	**R:**TGTTTTACCAAATAATTATTTTATTAGCAGATAAA
(antisense)	**F:**(T7) TGTTTTACCAAATAATTATTTTATTAGCAGATAAA
	**R:**GGCGCTCTGGGGCCGGGATCGGCGTCGGGG

Primers for antisense oligomer affinity pull-down assays

Lnc030 sense oligo DNA	5' (biotin-)AAAGCAAGGAAGTGGGACGC'3
Lnc030 antisense oligo DNA	5' (biotin-)TTCGAGTTCATGAAGAAGTTGC'3
SQLE sense oligo DNA	5' (biotin-) AACACGCGCAGACCTTCGTTG'3
SQLE antisense oligo DNA	5' (biotin-)ATCCAAATCTTGGTCCAAGAGTT'3
